# Controlling Intrinsic Quantum Confinement in Formamidinium Lead Triiodide Perovskite through Cs Substitution

**DOI:** 10.1021/acsnano.2c02970

**Published:** 2022-05-24

**Authors:** Karim A. Elmestekawy, Adam D. Wright, Kilian B. Lohmann, Juliane Borchert, Michael B. Johnston, Laura M. Herz

**Affiliations:** † Department of Physics, Clarendon Laboratory, University of Oxford, Parks Road, Oxford OX1 3PU, United Kingdom; ‡ Institute for Advanced Study, Technical University of Munich, Lichtenbergstrasse 2a, D-85748 Garching, Germany

**Keywords:** perovskites, mixed-cation perovskite, FAPbI_3_, quantum confinement, absorption coefficient, photoluminescence, time-resolved photoluminescence

## Abstract

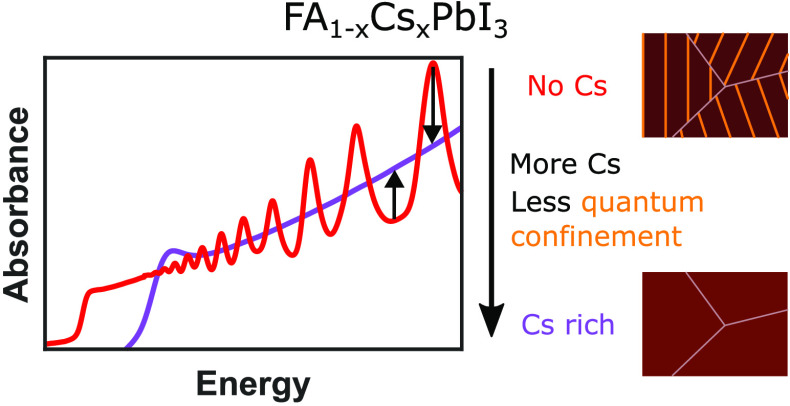

Lead halide perovskites are leading candidates for photovoltaic and light-emitting devices, owing to their excellent and widely tunable optoelectronic properties. Nanostructure control has been central to their development, allowing for improvements in efficiency and stability, and changes in electronic dimensionality. Recently, formamidinium lead triiodide (FAPbI_3_) has been shown to exhibit intrinsic quantum confinement effects in nominally bulk thin films, apparent through above-bandgap absorption peaks. Here, we show that such nanoscale electronic effects can be controlled through partial replacement of the FA cation with Cs. We find that Cs-cation exchange causes a weakening of quantum confinement in the perovskite, arising from changes in the bandstructure, the length scale of confinement, or the presence of δ_H_-phase electronic barriers. We further observe photon emission from quantum-confined regions, highlighting their potential usefulness to light-emitting devices and single-photon sources. Overall, controlling this intriguing quantum phenomenon will allow for its suppression or enhancement according to need.

Metal halide perovskites have emerged as promising materials for photovoltaic cells with reported power conversion efficiencies (PCEs) of single-junction cells improving from an initially reported 3.8%^1^ to over 25%^2^ in just over a decade. Their excellent properties include high charge-carrier mobilities,^3^ long diffusion lengths,^4,5^ low exciton binding energies,^6^ broadly tunable absorption spectra,^7^ and facile fabrication.^8^ In addition, promising light-emitting^9−13^ and lasing applications^14−16^ are rapidly emerging. In this context, perovskites with lowered electronic dimensionality are increasingly of interest, including two-dimensional perovskites^17,18,18−20^ and perovskite nanocrystals and quantum dots.^12,21−24^ Such nanostructured materials tend to exhibit electronic properties substantially different from those of their three-dimensional counterparts, including altered emission color and bandwidth,^22,23,23^ improved photoluminescence quantum yield,^25^ more easily achievable population inversion,^14−16^ and photon recycling.^17,24^ However, a major drawback is that nanostructured materials usually have additional complexities associated with their fabrication,^26^ making facile new fabrication methods a highly sought-after goal.

One promising route to the facile generation of nanostructured domains is through the use of formamidinium lead triiodide (FAPbI_3_), which has recently been shown to exhibit intrinsic electronic quantum confinement in a small subset of the volume of nominally bulk thin films.^27^ This surprising property manifests through sharp above-bandgap features superimposed on the bulk absorbance spectra of FAPbI_3_ films, which can be discerned in many published spectra,^28−31^ but had until recently gone unnoticed and unexplained. A recent report^27^ provided experimental evidence and theoretical simulations to demonstrate that these sharp absorption features arise from intrinsic quantum confinement on the length scale of ∼10 nm occurring in subvolumes of nominally bulk FAPbI_3_ films. However, the usefulness of intrinsic electronic confinement in FAPbI_3_ will depend critically on the targeted application. On the one hand, FAPbI_3_ in its three-dimensional cubic room-temperature phase (termed α-phase) is particularly well-suited for single-junction PV applications, with its bandgap of 1.55 eV close to the optimal theoretical value for monojunctions,^32^ and PCEs achieving a record value of 25.7% for cells based on FAPbI_3_.^33−35^ For such photovoltaic applications, confinement domains exhibiting barriers to charge motion will to some extent hinder the passage of photocurrent through the bulk and toward charge-extraction layers, lowering efficiencies. On the other hand, intrinsic quantum confinement may allow for facile fabrication of efficient light-emitting devices, if emission from such domains can be observed. With targeted fabrication and processing, such as strain engineering, this system thus offers the possibility of self-assembled quantum-confined states being readily formed without the need for a cumbersome fabrication process, making it an intriguing candidate for light-emitting diodes, lasers and displays, and single photon emitters for use in quantum computing.^14−16,36,37^

The above considerations show that control over intrinsic quantum confinement in FAPbI_3_ films is critical to its successful implementation in specific applications. We here attempt such control through partial substitution of the FA^+^ cation with the much smaller Cs^+^ cation, because it has recently been postulated that the origin of intrinsic quantum confinement in FAPbI_3_ could derive from the presence of trace amounts of the hexagonal nonperovskite δ_H_-phase of FAPbI_3_.^27^ Since the δ_H_-phase in its bulk form has an electronic bandgap near 2.3 eV, its presence even as thin layers will represent barriers to electronic motion through the black FAPbI_3_ perovskite α-phase, leading to charge carriers experiencing electronic confinement in quantum wells or periodic superlattices.^27,38^ Replacement of FA with Cs has been shown to stabilize the otherwise metastable α-phase of FAPbI_3_^28,39^ against deterioration into the δ_H_-FAPbI_3_ phase, which is the thermodynamically favored phase at room temperature.^40^ In general, A-cation engineering of ABX_3_ metal halide perovskites has proven highly effective at improving performance, structural stability,^41−44^ and crystallinity^29^ and suppressing phase transitions in the material.^3,43^ Specifically, the large size of the FA A-cation in FAPbI_3_ proves detrimental to its structural stability, which alloying with the much smaller Cs cation has been shown to remedy.^29,45,46^ Therefore, alloying with Cs may not only eliminate large crystallites of δ_H_-phase FAPbI_3_ but also potentially be able to suppress the smaller remnant inclusions that act as the potential barriers which lead to intrinsic quantum confinement in FAPbI_3_.

In this article, we report the control of intrinsic quantum confinement in FAPbI_3_ through partial A-cation substitution of FA with Cs. We probe the presence of such nanoscale domains through their impact on the electronic structure, evident from features superimposed on the absorption and emission spectra of the otherwise bulk semiconductor. We propose that alloying with Cs reduces quantum confinement owing to the interplay between changes in bandstructure and confinement length scale and the removal of trace layers of the hexagonal δ_H_-phase. We observe that Cs percentages, *x*, in excess of 40% are required for the complete removal of intrinsic quantum confinement effects in FA_1–*x*_Cs_*x*_PbI_3_, significantly higher than those commonly reported to suffice for structural stabilization of FAPbI_3_.^29,40,47−50^ Furthermore, we report a series of emission peaks with photon energy significantly above the bulk bandgap in FAPbI_3_, which we show to be associated with radiative recombination of intrinsically confined charge carriers. Overall, we demonstrate that the degree of intrinsic nanoscale confinement in FA_1–*x*_Cs_*x*_PbI_3_ may thus be controlled through simple stoichiometric engineering rather than intensive nanoscale processing.

## Results and Discussion

Absorption spectra of solution-processed FAPbI_3_ films reported in the literature often exhibit the peculiar absorption features being investigated in this study,^28,43,50−53^ though this is not typically commented on. However, to ensure surface uniformity and controllable composition, we based our investigation on vapor-deposited FAPbI_3_ films of 100 nm thickness in which the partial substitution of FA with Cs had been induced through a solution-based cation exchange. FAPbI_3_ films were evaporated onto either quartz or sapphire substrates through thermal coevaporation of FAI and PbI_2_^54−56^ and subsequently dipped for three seconds in IPA solutions of differing cesium acetate (CsCH_3_COO) concentrations, following the procedure outlined by Jiang et al.^57^ Two control films were used for this study: an undipped neat FAPbI_3_ film and one dipped in an IPA solution. Five other FAPbI_3_ films were subject to A-cation exchange through dipping in IPA solutions of 0.2, 0.5, 1, 5, and 7.5 mg/mL CsCH_3_COO concentrations. Further details on the fabrication method of the thin films are described in Section 1 in the SI.

**Figure 1:**
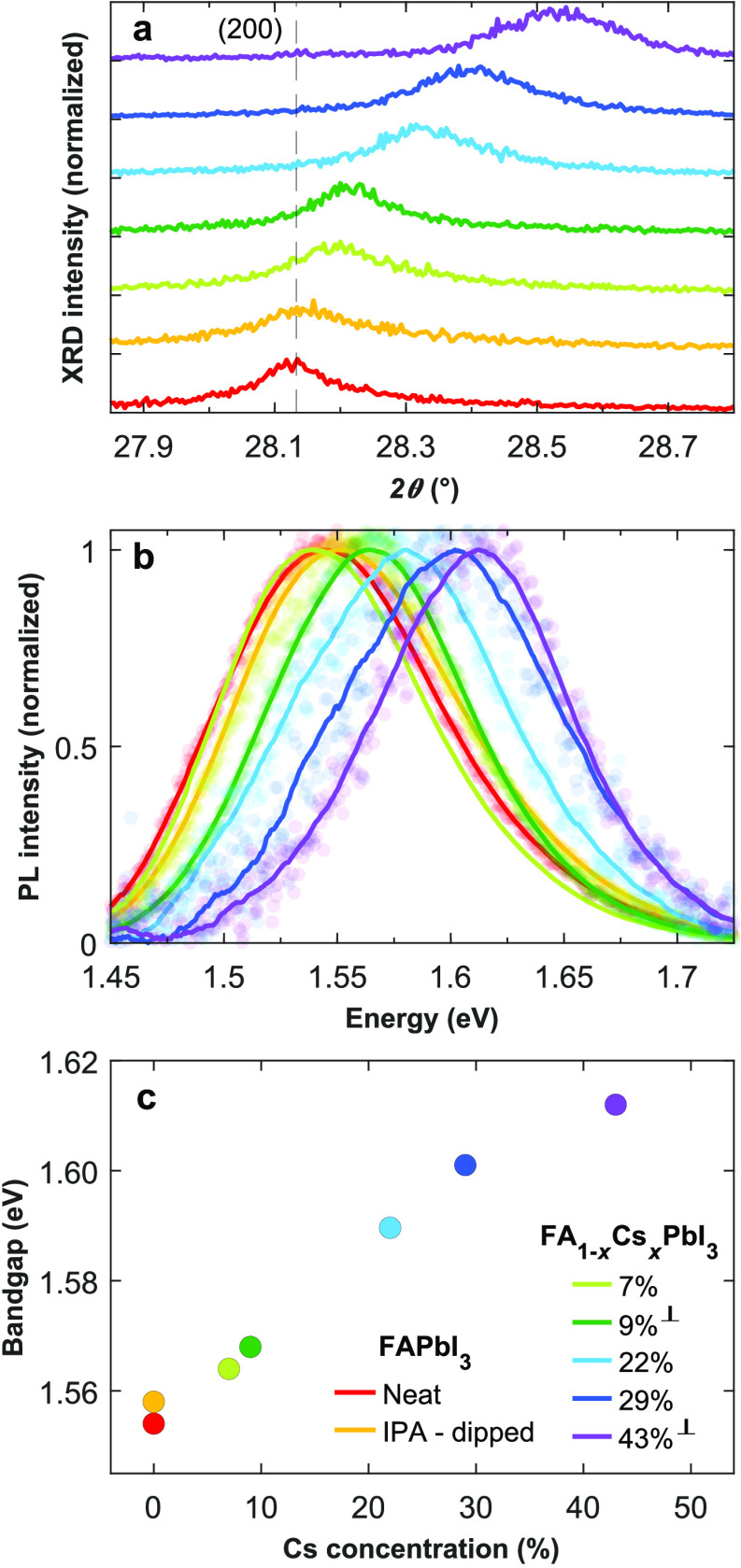
Structural and optical characterization of FA_1–*x*_Cs_*x*_PbI_3_ films. (a) Excerpts from recorded XRD patterns centring on the pseudocubic (200) peak of FA_1–*x*_Cs_*x*_PbI_3_. The vertical dotted line represents the (200) peak position of the FAPbI_3_ neat film. For visual clarity, the XRD patterns are successively vertically offset. (b) Normalized, smoothed, room-temperature PL spectra of the FA_1–*x*_Cs_*x*_PbI_3_ films represented by solid lines, while the raw data are represented by the scatter points. (c) Relationship between the optical bandgap extracted from Elliott fits to the absorption onset and the Cs content *x* evaluated based on the XRD peak positions (as described in SI Figure S4 and Section 2 in the SI). The legend in (c), also applies to (a), and (b).

We begin by determining the extent to which Cs has replaced FA in the as-grown FAPbI_3_ films as a result of dipping in CsCH_3_COO solution. Figure 1 shows the effects of Cs incorporation on the X-ray diffraction (XRD) patterns, photoluminescence (PL) spectra, and bandgap determined from absorption onsets. We note that since quantum confined domains in these films tend to occupy only a few percent of the total film volume,^27^ the recorded spectra mostly reflect bulk FA_1–*x*_Cs_*x*_PbI_3_ properties. The shifting of the (200) cubic FAPbI_3_ peak toward higher 2θ angles with higher CsCH_3_COO concentrations is indicative of a crystal lattice contraction in accordance with Cs^+^ ions replacing the bulkier FA^+^ in the perovskite.^46^ We utilize these crystallographic trends to determine the spatially-averaged incorporated Cs percentage from the linear relationship between the extracted pseudocubic lattice parameter of the films and their Cs content, as outlined previously by Jiang et al.^57^ In their work, the authors correlated the peak positions observed in the XRD patterns with Cs percentages determined near the film surface and spatially averaged across the film through structural characterization techniques such as X-ray fluorescence and X-ray photoelectron spectroscopy for FA_1–*x*_Cs_*x*_PbI_3_ films produced via the CsCH_3_COO dipping method we replicated here. Based on their findings, we determine here that the FAPbI_3_ films dipped in IPA solutions of CsCH_3_COO of concentrations 0.2, 0.5, 1, 5, and 7.5 mg/mL resulted in FA_1–*x*_Cs_*x*_PbI_3_ films with spatially averaged 7%, 9%, 22%, 29%, and 43% Cs content, respectively. Further details for the calibration method are provided in Section 2 in the SI, which also shows full XRD patterns in SI Figure S1. We further note that this method of Cs incorporation through A-cation exchange leads to a certain nonuniformity in the distribution of Cs content throughout the thickness of the film as observed previously by Jiang et al.^57^ and apparent from the broadening of the XRD peaks with increasing Cs content (see Figure 1a for XRD peaks and SI Figure S3a for extracted values of peak widths). We note that the δ_H_-phase of FAPbI_3_ is not particularly apparent in the XRD patterns for all films except for that of the neat (undipped) FAPbI_3_ film. XRD patterns magnified around the typical (100) peak of the δ_H_-phase of FAPbI_3_ at 2θ = 11.8°^39^ (see SI Figure S2) show little diffraction signal, which is consistent with any incorporated electronic barriers arising from δ_H_-phase inclusions being relatively thin, as had previously been postulated.^27^

The incorporation of Cs resulting from dipping of FAPbI_3_ films in CsCH_3_COO solutions is also evident from blue-shifts in the room-temperature PL spectra (Figure 1b) and the bandgap energies (Figure 1c) in accordance with general observations for FA_1–*x*_Cs_*x*_PbI_3_ with increasing *x*.^28,58^ The bandgap values were determined through fitting of absorption coefficient onsets with Elliott theory, which accurately accounts for both the excitonic feature and Coulombic contribution to the absorption continuum.^59,60^ A simpler procedure based on commonly used Tauc fits^29,45,58^ yields similar trends (see SI Figure S8) but is somewhat less accurate, in particular for polycrystalline films.^61^ We note that a direct determination of Cs content from optical spectra rather than XRD patterns is however cumbersome, as the linear relationship generally expected to hold between the pseudocubic lattice parameters of a set of materials and their alloying fraction (Vegard’s law) does not necessarily translate to a linear relationship for the electronic bandgap, as indeed we see in Figure 1c. Bandgap values relatively rarely relate to a weighted mean of their constituents’ values leading to the well-known effect of “bandgap bowing”,^58,62,63^ which in the case of FA_1–*x*_Cs_*x*_PbI_3_ may, for example, arise from significant ionic size mismatch between the FA^+^ and Cs^+^ ions inducing strain in the material.^3,43,50^

**Figure 2:**
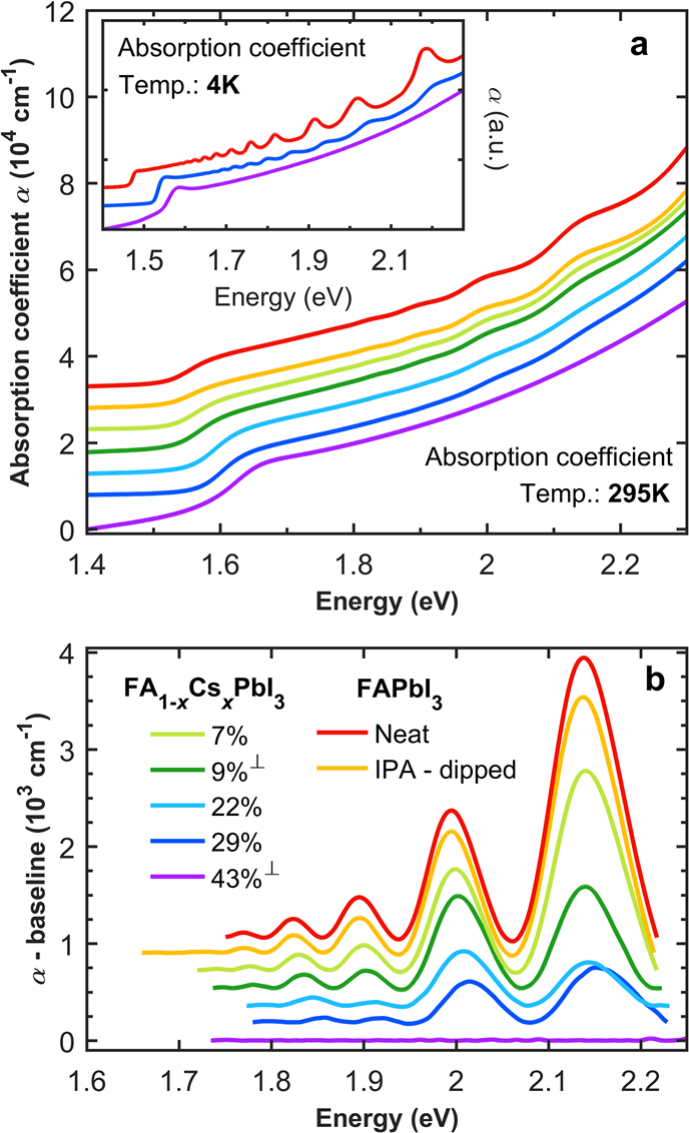
Absorption coefficient spectra and decoupled peaks. (a) Absorption coefficient spectra of FA_1–*x*_Cs_*x*_PbI_3_ films with different Cs content *x* at room temperature (295 K, main panel) and 4 K (inset). (b) Peak features decoupled from the rest of the bulk-like absorption spectra at room temperature. For visual clarity, the absorption coefficient spectra and peak features are successively vertically offset by 0.5 × 10^4^cm^–1^ and 0.15 × 10^3^cm^–1^ respectively. The legend in (b) also applies to (a). Films are evaporated on either quartz or sapphire (indicated by ⊥) substrates, indicating that trends are independent of substrate choice.

The absorption coefficient spectra of FA_1–*x*_Cs_*x*_PbI_3_ films clearly reveal that the incorporation of Cs affects the prevalence of domains exhibiting quantum confinement. Figure 2 shows that the room-temperature absorption coefficient spectra display above-bandgap peak features whose prominence declines with increasing Cs content. To visualize these features more readily, they were decoupled from the spectrum using a phenomenological spline baseline connecting all the troughs of the features together, and subtracting this baseline from the measured absorption spectra produces the plot in Figure 2b (full details of method provided in Section 4.3 of the SI). As Cs replaces FA, two trends are clearly apparent: The band-edge blue-shifts, as incorporation of the smaller Cs increases the bandgap (Figure 1), and the prominence and widths of the peak features gradually decline until they effectively disappear for the FA_1–*x*_Cs_*x*_PbI_3_ film containing 43% Cs. These observations therefore demonstrate conclusively that introducing Cs into the perovskite stoichiometry can reduce and control intrinsic quantum confinement.

**Figure 3:**
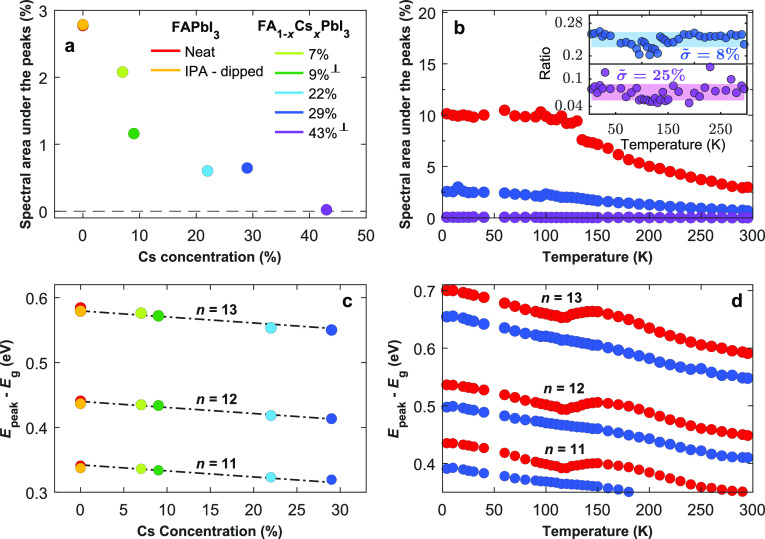
Quantitative analysis of the prominence of absorption peak features, and the derived energy of quantum confinement. (a, b) Spectral area underneath the absorption peaks, given as a percentage of the total area under the absorption coefficient spectrum, used to parametrize the occurrence of quantum confinement: (a) Values at 295 K as a function of Cs content *x* in FA_1–*x*_Cs_*x*_PbI_3_ and (b) values as a function of temperature for the compositions: FAPbI_3_ (red circles), FA_0.71_Cs_0.29_PbI_3_ (blue circles), and FA_0.57_Cs_0.43_PbI_3_ (purple circles). The inset in (b) shows the spectral areas under the absorption peaks for FA_0.71_Cs_0.29_PbI_3_ (blue circles) and FA_0.57_Cs_0.43_PbI_3_ (purple circles) divided by the values for FAPbI_3_, indicating a temperature-independent ratio (within a standard deviation of the mean, as visualized with the shaded areas). The value of σ̃ shown indicates the standard deviation divided by the mean value, parametrizing the spread. (c, d) Confinement energy, calculated as the difference between the absorption peak position *E*_peak_ and the bandgap *E*_g_ extracted from Elliott fits to absorption onsets: (c) The confinement energy extracted from peaks with index numbers *n* = 11–13 at room temperature as a function of Cs content across the FA_1–*x*_Cs_*x*_PbI_3_ film series, with lines showing a global linear fit of identical gradient yielding 0.93 meV decline per Cs percent added, and (d) the confinement energy as a function of temperature for FAPbI_3_ (blue circles) and FA_0.71_Cs_0.29_PbI_3_ (red circles). The legend in (a) also applies to (b–d). Films are evaporated on quartz or sapphire (indicated by ⊥) substrates.

We note that the almost-complete elimination of quantum confinement features for sufficiently high Cs content (43%) is also evident in the low temperature (4 K) absorption spectrum (see inset in Figure 2a, and SI Figure S11 for decoupled peaks). This absence is particularly interesting because such features have previously been shown^27^ to be enhanced with reduction in temperature (as also evident from Figure 2a). Our determination of the absorption coefficient spectra and decoupled peaks across a range of different temperatures for the neat FAPbI_3_ film and FA_1–*x*_Cs_*x*_PbI_3_ films with 22%, 29%, and 43% Cs (SI Figures S10 and S11, respectively) shows that this trend of enhanced features with lowered temperature clearly holds for a range of Cs contents. Such enhancement of quantum confinement at reduced temperature may arise from lattice contraction and subsequent reduction in confinement length scale and a decrease in electron–phonon coupling and thermal fluctuations, which may strengthen the quantum confinement, enhancing the amplitude and reducing the width of the associated peaks, making them clearly discernible.^27^ In addition, the increased prominence may derive from a growth in the material volume experiencing confinement as δ_H_-phase formation becomes more prominent at lower temperatures.^40^ However, to narrow down the origin of this enhancement of quantum confinement, more in-depth quantitative analysis is required. To that end, we first define and calculate from our data two useful parameters. First, we define the spectral area under the peaks as the area between the experimental absorption coefficient spectrum and the spline baseline connecting the troughs (i.e., the integral over curves such as those shown in Figure 2b), stated as a percentage of the overall area under the absorption spectrum. Such data indicate a relative prominence of the features in the absorption spectra and are displayed in Figure 3a,b for different Cs contents and temperatures. Second, we calculate the confinement energy for a given absorption peak feature by simply subtracting from each peak energy *E*_peak_ the bandgap value *E*_g_ previously extracted from Elliott fits (Figure 1c). Subsequent peaks were numbered incrementally toward higher energy, with peak index value *n* = 0 referring to the lowest-energy discernible peak in the absorbance spectra for a FAPbI_3_ film at 4 K.^27^Figure 3c,d display the changes in confinement energy *E*_peak_ – *E*_g_ for different Cs content and temperatures. Section 4.3 in the SI provides further details on the peak indexing method, definitions, and extraction of both parameters.

We first explore how substitution of FA with Cs affects the confinement energy *E*_peak_ – *E*_g_. As Figure 3c indicates, increasing Cs content in FA_1–*x*_Cs_*x*_PbI_3_ leads to a decline in confinement energy, a trend opposite to that of the blue-shift of the bandgap. We have previously postulated that intrinsic quantum confinement in FAPbI_3_ may be linked with the spontaneous formation of electronic barriers in the material deriving from relatively thin, and potentially periodic inclusions of δ_H_-phase, or possibly ferroelectric domain walls.^27^ We further showed that the quadratic dependence of the confinement energy on the peak index was compatible with energy levels deriving from a superlattice structure (Krönig–Penney model) whose energy levels may approach those of an infinite quantum well. As discussed in Section 5 in the SI, in this approximation, the confinement energy depends on the square of the peak index (∝ *n*^2^) and is inversely proportional to the electron–hole reduced effective mass (∝ 1/μ*) and the square of the length scale (or periodicity) of electronic confinement (∝ 1/*L*^2^). By fitting, as a crude first-order approximation, a global linear regression to the confinement energy *E*_peak_ – *E*_g_ at room temperature for peak indices *n* = 11–13 (see Figure 3c), we determine a gradual decline in confinement energy by 0.93 meV per Cs percent added, up until 29% Cs content. These linear fits indicate that within this Cs content range, the confinement energy falls by 27 meV, corresponding to a mean fractional fall of around 6% across the *n* = 11−13 peaks. Based on the known trends in the literature for a lattice contraction and an increase in the electron–hole reduced effective mass μ* with increasing Cs content for FA-based perovskites, such a decline in confinement energy with increasing Cs content could thus be caused by a combination of a small decrease in the confinement length scale *L* and a considerably larger counteracting increase in the reduced effective mass. Our analysis of the XRD patterns provided in Table S1 of SI Section 2 indicates a fractional reduction in lattice parameter by 0.92% between 0% and 29% Cs content, which would induce an analogous relative contraction of the associated intrinsic confinement length scales. Similarly, the electron–hole reduced effective mass at low temperatures has been shown to increase from FAPbI_3_ to CsPbI_3_ from 0.09*m*_e_^64^ to 0.114*m*_e_,^65^ respectively, as expected from changes to the electronic band structure.^57,58,65−67^ Assuming a linear relationship for the change in reduced effective mass with Cs concentration,^64,65^ the fractional increase in μ* would correspond to 7.73% between 0% and 29% Cs concentration films. Therefore, this percentage increase in μ* (by 7.73%) is more than sufficient to counterbalance the reduction in confinement energy caused by a decline in *L*^2^ (by 2 × 0.92%) accompanying the increase in Cs content, which, taken together, would be expected to decrease the confinement energy (∝1/(μ*L*^2^)) by ∼5.4%, similar to what is actually observed in Figure 3c.

As a second step, we proceed by analyzing the extent to which Cs incorporation affects the prevalence of peak features in the absorption of the FA_1–*x*_Cs_*x*_PbI_3_ films. First, we note that the spectral area under the peaks (Figure 3a) and the peak amplitude (Figure 2) gradually decrease with increasing Cs content until they mostly vanish for the film with 43% Cs content, commensurate with the almost-complete elimination of quantum confinement effects. The accompanying gentle decline in confinement energy discussed above is clearly too small to be the main cause of the fading peak features in the absorption spectra. We therefore propose that instead, much of the amplitude decline is caused by a reduction in the volume fraction supporting such quantum confined domains. This hypothesis is also supported by the observation of a constant ratio of peak amplitudes for films with different Cs content as a function of temperature (see inset in Figure 3b), suggesting that the films of different Cs content share a mechanism of amplitude decline, whose temperature dependence is common between them.

We propose two possible reasons why substituting FA for Cs may cause fewer confinement domains to be present in the film volume. First, we note that incorporation of Cs into FAPbI_3_ has been shown to reduce the formation of large δ_H_-phase domains (apparent in literature XRD patterns with (100) peak around 2θ = 11.8°)^39^ for addition of as little as 5% Cs.^29,40,47−50^ We have previously postulated that periodic inclusions of thin layers of δ_H_-phase, for example, as a result of strain, may lead to the quantum confinement effects observed in FAPbI_3_.^27^ Such thin electronic barriers with widths of <10% of the well width are capable of inducing prominent effects in the absorption spectrum^27^ while not necessarily generating noticeable XRD diffraction amplitude at 2θ = 11.8°. We note that the amount of Cs content required to almost entirely remove quantum confinement effects (≥40%) is significantly larger than that (∼5%)^29,40,47−50^ needed to eliminate extended domains of bulk crystalline δ_H_-phase, which may relate to their differing thermodynamics of formation. An alternative reason for the elimination of intrinsic quantum confinement for high Cs content in FA_1–*x*_Cs_*x*_PbI_3_ may derive from Cs carrying no net dipole moment. The FA^+^ cation in FAPbI_3_ is associated with a significant dipole moment that may potentially support ferroelectric effects.^27^ While ferroelectricity in hybrid metal halide perovskites is still a contentious topic,^68−70^ ferroelectric domain walls may also present electronic boundaries that could induce quantum confinement. However, if intrinsic quantum confinement in FAPbI_3_ could result from ferroelectric domain boundaries, as has been postulated previously,^27^ then the addition of the spherically symmetric Cs has the potential to disrupt such effects.

Our observations clearly demonstrate that Cs substitution in FAPbI_3_ is able to control the presence of domains exhibiting intrinsic quantum confinement, leading to their almost-complete elimination at high Cs content. Alternatively, this same level of control could potentially be achieved through the incorporation of different additives or through strain engineering or templating during the fabrication process, which has in the past been shown to affect the formation of δ_H_-phase in FAPbI_3_.^40,41,71−73^ An argument could then be made that quantum confinement in FAPbI_3_ could instead also be enhanced, possibly through strain engineering or nucleation agents, capable of controlling and stabilizing the self-assembled nanostructures. Indeed, absorption and PL features of lower-dimensional phases associated with MAPbI_3_ films were previously reported to have been enhanced and stabilized using stoichiometric and additive engineering.^74^ Such enhancement of nanoscale effects through facile bulk processing techniques could provide a future pathway for efficient light emission and single-photon sources for quantum applications. To test the tantalizing possibility of emission emerging from these nanostructures (i.e., from electronically confined states), we performed PL measurements on FAPbI_3_ at low temperatures, for which nonradiative recombination is suppressed because of thermal depopulation of the available phonons to mediate such recombination^75,76^ and for which radiative recombination is enhanced as a result of the sharpening of the Fermi−Dirac distribution function.^60,77^

**Figure 4:**
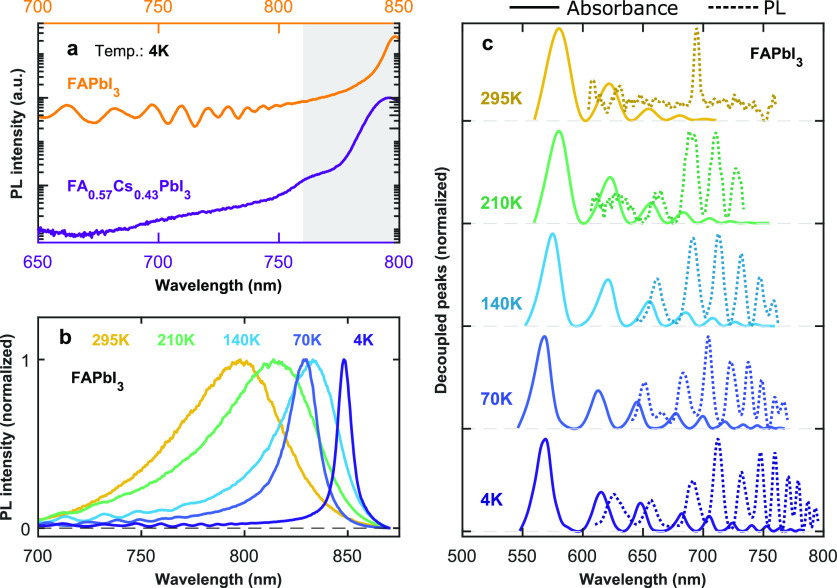
PL spectra. (a) PL spectra on a log scale of an FAPbI_3_ (IPA dipped) and a FA_0.57_Cs_0.43_PbI_3_ film at a temperature of 4 K showcasing the presence and absence, respectively, of above-bandgap peak features. The two spectra are vertically offset for visual clarity. (The full PL spectra of both films on a linear scale are shown in SI Figure S19 and discussed in SI Section 6.2.) (b) Temperature dependence of the normalized PL spectra for an FAPbI_3_ film (IPA-dipped control). (c) Normalized spectra of the decoupled absorption and emission peak features for the FAPbI_3_ film, recorded at different temperatures. Peak features were decoupled from the absorption (represented by solid lines) and PL (represented by dotted lines) spectra using a spline phenomenological baseline fitting method in both cases. The PL spectra are collected following excitation by a 398 nm wavelength diode laser under continuous-wave operation.

Notably, we are indeed able to observe PL peak features at energies above the bandgap for FAPbI_3_ at 4 K, at energetically similar positions to those observed in the absorption coefficient spectra. As shown in Figure 4a, such features are weak but clearly apparent in an IPA-dipped FAPbI_3_ film; however, they disappear when 43% of FA has been substituted for Cs in FA_0.57_Cs_0.43_PbI_3_. Such dependence on Cs incorporation is similar to that observed for the absorption peak features, leading us to attribute these observed PL peak features to the same quantum phenomenon. It therefore appears that a subset of photogenerated charge carriers spends sufficient time in quantum-confined domains to yield emission from the associated states.

To further explore the fundamental physics behind the PL peak features and the quantum confinement, we examine the temperature dependence of the emission from quantum confined domains. Figure 4b shows that the PL peak features gradually broaden and progressively become less discernible from the underlying main PL spectrum as the temperature increases, similar to the temperature trends observed in the absorbance spectra (see SI Section 6.1 for further discussion). To quantitatively analyze this observation and unmask the relationship between these features in both the PL and absorbance spectra, we decoupled the PL peak features from the underlying spectra in a manner analogous to that utilized for the absorption spectra (see SI Figures S15 and S9). The overlaid decoupled peaks from the PL and absorbance spectra (shown in Figure 4c) clearly demonstrate that these indeed occupy the same energetic range and similar wavelength position patterns. However, two clear differences are worth noting. First, a slight Stokes shift is evident between absorption and emission peaks. Such shifts may arise, for example, from relaxation of charge carriers within the confined bands that emerge from the Krönig–Penney model or within the slightly disordered density of states available in a domain, evidenced by the nonzero broadening that is present even at low temperature and may derive, for example, from well width fluctuations. The apparent Stokes shift may also partly be related to photon reabsorption effects, though we note that such reabsorption alone cannot explain the modulations observed in the PL spectra, as discussed in detail in Section 6.1 in the SI and visualized in SI Figure S14. Second, and more importantly, the peak amplitude trends with energy markedly differ between the absorption and emission features. For the absorption peaks, the amplitude progressively increases with higher energy (shorter wavelength), as observed previously and attributed to a higher density of states available at higher energies that generate strong transition densities.^27^ For the PL peaks, however, an opposite trend is observed with the lower-energy confined states having the largest PL amplitude. This crossover in trends is most likely caused by fast charge-carrier relaxation from higher-energy to lower-energy quantum-confined states. We note that such relaxation through the internal states of a quantum structure is generally relatively rapid, such that the observation of emission from higher-lying states depends on a competitive trade-off with the time scale for radiative recombination.

We note that emission from higher-lying quantum confined states has been observed before for zero-dimensional quantum dots,^78−82^ which typically exhibit a spectral dependence on excitation intensity owing to ground-state filling and hot phonon bottlenecks. We, however, do not observe such an excitation intensity dependence (see SI Figure S16). Instead, the observed emission could be a result of some moderate phonon bottleneck slowing down the relaxation dynamics, with fast funneling out of the confined domains into the bulk phase and radiative recombination competing on the same time scale as this internal relaxation. This is further discussed along with fitting models for the PL confinement energy in Section 6.1.1 of the SI.

**Figure 5:**
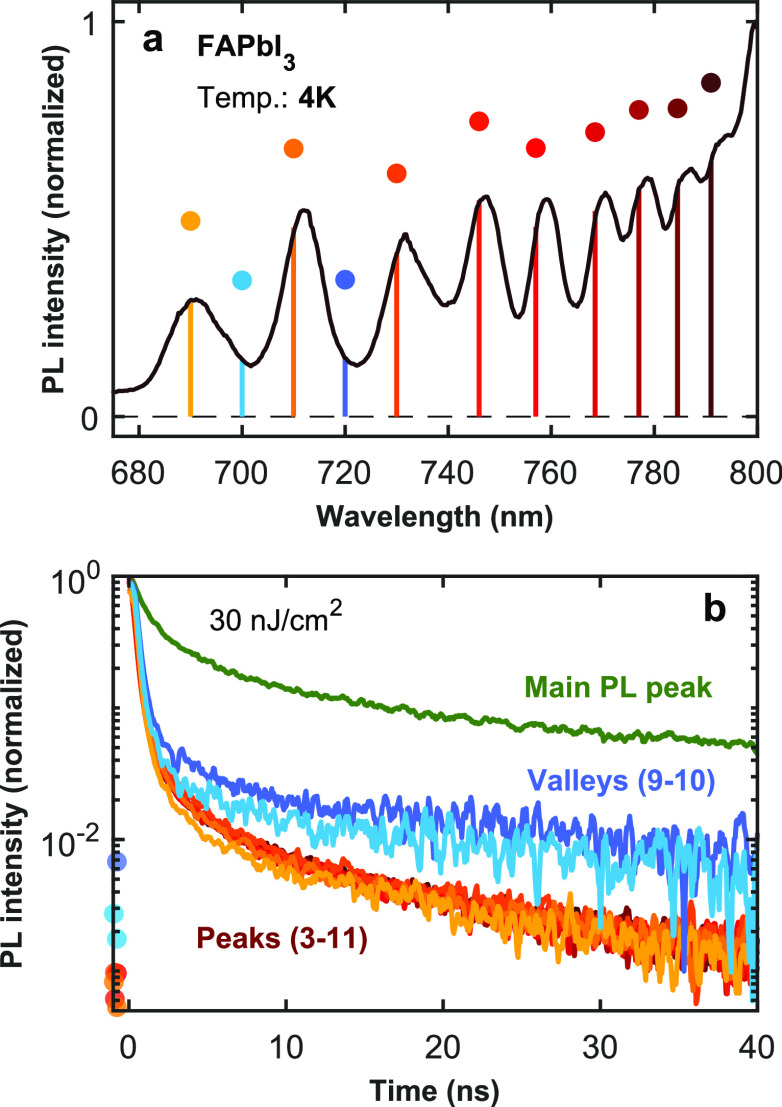
TRPL of FAPbI_**3**_. (a) High-energy (short-wavelength) tail of the PL spectrum of FAPbI_3_ (IPA-dipped film) at 4 K. (b) TRPL decay traces recorded at the spectral positions indicated with colored dots and solid vertical lines in the spectrum shown in (a). The PL decay traces were recorded following an excitation by a 398 nm picosecond pulsed diode laser at a repetition rate of 1 MHz for the main peak of the PL spectrum and a repetition rate of 10 MHz for the peaks and valleys highlighted in (a). The numbering of the peaks and valleys were such that the lowest-energy discernible peak and valley were indexed peak #0 and valley #0, respectively.

The above observations suggest that significant early time relaxation may occur in quantum-confined states of FAPbI_3_ following photoexcitation. Charge carriers may relax internally within a domain exhibiting quantum confinement, either through interlevel relaxation or diffusion to lower-energy parts of the domain. They may also funnel away entirely from quantum-confined regions and into the bulk. To probe for such effects, we measured TRPL at low temperature (4 K) with 40 ps time resolution for different spectral emission regions, as shown in Figure 5 (analogous data for IPA-dipped FAPbI_3_ at room temperature and FA_0.57_Cs_0.43_PbI_3_ at 4 K are shown in SI Figures S20 and S21, respectively). We find that while the main PL peak shows a dispersive decay transient over tens of nanoseconds, the emission arising from the peaks associated with quantum confinement is substantially more rapid. This initial rapid component is beyond our time-resolution (see SI Figure S22) and most likely associated with internal relaxation within the domain but potentially also accelerated by enhanced exciton formation, given that exciton binding energies are enhanced by quantum confinement.^83−87^ We note that PL transients recorded in between peaks (i.e., in the valleys) show intermediate behavior to that of the peaks and bulk emission, most likely owing to limitations on the spectral resolution of our experimental setup. The slow dynamics observed at long times after excitation may therefore mostly be associated with very weak high-energy tails of the bulk emission. Overall, these PL data paint a conclusive picture of quantum emitters being present within regions surrounded by bulk-like FAPbI_3_.

## Conclusions

Overall, we have shown successful control over the strength of intrinsic quantum confinement exhibited by FAPbI_3_ perovskite and its derivatives. We have demonstrated that Cs addition allows for the gradual elimination of such confinement effects, which disappear almost entirely as the Cs content in FA_1–*x*_Cs_*x*_PbI_3_ reaches 43%, both at room and low temperature. We postulate that this reduction mostly derives from a decrease in the volume fraction hosting electronic boundaries that cause quantum confinement. We suggest that when such domain walls are caused by either δ_H_-phase inclusions or ferroelectric effects, Cs addition may mitigate their formation through either structural effects or its nonpolar nature, respectively. We further find that Cs incorporation has a moderate impact on the confinement energy, which gradually reduces by ∼6% from FAPbI_3_ (0% Cs) to FA_0.71_Cs_0.29_PbI_3_ (29% Cs) when averaged over the *n* = 11−13 peaks, owing to competing changes in effective reduced electron–hole mass and the lattice spacing that defines the length scales of confinement. Our observations of the first clear emission features from such quantum-confined domains in FAPbI_3_ reveal features comparable to those found in the corresponding absorption spectra. However, a comparison of emission and absorption spectra and an analysis of the PL decay transients reveal that charge carriers are likely to undergo rapid relaxation following the initial excitation. Fast initial dynamics are most likely associated with internal charge-carrier relaxation within a given confinement domain, followed by funneling from such domains into the bulk phase. Overall, our study demonstrates that such intrinsic quantum confinement effects can be controlled depending on the desired application. While for photovoltaics devices, charge-carrier extraction may be impeded by the presence of domain walls supporting quantum confinement, for light-emitting applications, such confinement will be advantageous, making such easily fabricated, self-assembled periodic nanostructures highly desirable. Our work allows for an understanding of the underlying mechanisms influencing quantum confinement in FAPbI_3_ and FACs mixed-cation perovskite thin films, showing that full elimination of the above-bandgap features through Cs alloying is possible. Further exploring these features and methods of their control, for example, through strain engineering, tuning crystallization, or multiple A-cation alloying, will be instrumental in fully stabilizing quantum confinement and maximizing its utility and potential, for example, for light-emitting applications.

## Methods and Experiments

### Sample Fabrication

Lead(II) iodide PbI_2_ (Ultradry 99.999%, metals basis) and formamidinium iodide FAI (Dyesol, GreatCellSolar Materials) were heated and evaporated in separate crucibles in a modified Kurt J. Lesker dual source evaporation system, in a chamber pressure of 10^–6^ mbar. The evaporation temperature for FAI was 150 °C, and PbI_2_ was evaporated at 300 °C. The vapors condensed on rotating substrates to ensure uniform coating. The evaporated FAPbI_3_ films were then annealed on a hot plate at 170 °C for 3–5 min to ensure the formation of the photoactive perovskite α-FAPbI_3_ phase. Cesium acetate (CsCH_3_COO) (99.9% purity, Sigma-Aldrich) was dissolved in IPA to make dipping solutions with differing CsCH_3_COO concentrations (0.2, 0.5, 1, 5, and 7.5 mg/mL). Following the procedure outlined by Jiang et al.,^57^ we dipped the films in the prepared solutions for 3 s to induce cation exchange between the FA^+^ and Cs^+^ ions, dried the films with a N_2_ gun, and proceeded with annealing the dipped films on a hot plate at 170 °C for a further 20 min to ensure the complete evaporation of the FACH_3_COO byproduct from the dipping process. Solution preparation, film annealing, and storage between measurements were all done in a N_2_-filled, moisture- and O_2_-controlled glovebox.

### XRD Measurements

The XRD patterns were measured in air using a Panalytical X’pert powder diffractometer with a copper X-ray source (Cu–Kα X-rays with a wavelength of 1.5418 Å).

### Absorption Measurements

Reflectance (*R*) and transmittance (*T*) spectra were measured using a Fourier transform infrared spectrometer (Bruker Vertex 80v), configured with a tungsten halogen lamp illumination source, a CaF_2_ beamsplitter, and a silicon detector.

### Low-Temperature Measurements

The samples were all mounted in a gas-exchange helium cryostat (Oxford Instruments, OptistatCF2) in a helium atmosphere for the room-temperature measurements (where the outer vacuum chamber was pumped down to low pressures (<5 × 10^–5^ mbar)) and for the temperature-dependence study, where the temperature was varied between 4 K and 295 K in either 5 K or 10 K increments.

### Photoluminescence

A 398 nm diode laser (PicoHarp, LDH-D-C-405M) was used to photoexcite the samples, on a continuous wave setting at an intensity of 75.5 mW/cm^2^. The resultant PL was collected and coupled into a grating spectrometer (Princeton Instruments, SP-2558), which directed the spectrally dispersed PL onto a silicon iCCD (intensified charge coupled device, PI-MAX4, Princeton Instruments). The samples were mounted in a vacuum cell under low pressure (∼10^–2^ mbar).

TRPL of the thin films was measured using time-correlated single photon counting (TCSPC) following excitation by the 398 nm picosecond pulsed diode laser at a repetition rate of 1, 5, or 10 MHz (PicoHarp, LDH-D-C-405M). The resultant PL was collected and coupled into a grating spectrometer (Princeton Instruments, SP-2558), which directed the spectrally dispersed PL onto a photon-counting detector (PDM series from MPD), whose timing was controlled with a PicoHarp300 TCSPC event timer.

## Supporting Information Available

Fabrication method, experimental details, XRD patterns and analysis, additional absorption coefficient spectra and details on their fits, theoretical models on quantum confinement, additional steady-state and TRPL data, and the instrument response function for the TCSPC traces (PDF)
