# Supplementary material for: Controlling Intrinsic Quantum Confinement in Formamidinium Lead Triiodide Perovskite through Cs Substitution
Source: ACS Nano. 2022 May 24;16(6):9640–50. doi: 10.1021/acsnano.2c02970 (PMC9245356; doi:10.1021/acsnano.2c02970)
Supplement: Supplementary file 1 — nn2c02970_si_001.pdf [file nn2c02970_si_001.pdf]

Supporting Information:

Supporting Information: Controlling Intrinsic Quantum Confinement in Formamidinium Lead Triiodide Perovskite through Cs Substitution

Karim A. Elmestekawy,† Adam D. Wright,† Kilian B. Lohmann,† Juliane Borchert,† Michael B. Johnston,† and Laura M. Herz*,†,‡

†*Department of Physics, University of Oxford, Clarendon Laboratory, Parks Road, Oxford OX1 3PU, United Kingdom*

‡*Institute for Advanced Study, Technical University of Munich, Lichtenbergstrasse 2a, D-85748 Garching, Germany*

E-mail: laura.herz@physics.ox.ac.uk

Contents

1 Sample fabrication **S-3**

2 X-ray diffraction **S-4**

2.1 XRD spectra . . . S-4

2.2 Determining the Cs % of the films. . . . S-8

3 Steady-state photoluminescence **S-12**

4 Absorption measurements **S-14**

4.1 Elliott fitting . . . S-16

4.2 Tauc plots . . . S-19

4.3 Baseline fitting and peak indexing . . . S-21

4.4 Temperature-dependent study of the absorbance spectra . . . S-23

5 Theoretical models **S-25**

5.1 Infinite quantum well . . . S-25

5.2 Krönig-Penney potential . . . S-27

6 Temperature-dependent study of steady-state PL **S-27**

6.1 IPA-dipped $FAPbI_3$ . . . S-27

6.1.1 Confinement energy . . . S-35

6.2 $FA_{0.57}Cs_{0.43}PbI_3$ . . . S-37

7 Time-resolved PL (TRPL) **S-39**

Instrument Response Function . . . S-41

References **S-42**

1 Sample fabrication

In a modified Kurt J. Lesker dual source evaporation system, lead (II) iodide PbI_2 (Ultradry, 99.999%, metals basis) and formamidinium iodide FAI (Dyesol, GreatCellSolar Materials) were heated in separate crucibles until each of them evaporated. During evaporation the pressure in the chamber was 10^{-6} mbar. The evaporation temperature for FAI was 150°C, and PbI_2 was evaporated at 300°C. The vapors condensed on rotating substrates to ensure uniform coating. The evaporated $FAPbI_3$ films were then annealed on a hot plate at 170°C for 3-5 minutes to ensure the formation of the photo-active perovskite α-$FAPbI_3$ phase, which was visually confirmed by the change in the films' physical appearance to the expected black color. The films have a thickness of 100 nm as measured using a Bruker Dektak XTL Stylus profilometer. Cesium acetate ($CsCH_3COO$) (99.9% purity, Sigma-Aldrich) was dissolved in IPA to make dipping solutions with differing $CsCH_3COO$ concentrations (0.2, 0.5, 1, 5, and 7.5 mg/mL). Following the procedure outlined by Jiang et al.,[S1] we dipped the films in the prepared solutions for 3 seconds to induce cation exchange between the FA^+ and Cs^+ ions, dried the films with a N_2 gun, and proceeded with annealing the dipped films on a hot plate at 170°C for a further 20 minutes to ensure the complete evaporation of the $FACH_3COO$ byproduct from the dipping process. Solution preparation, film annealing and storage between measurements were all done in a N_2-filled, moisture-, and O_2-controlled glove box. The films were always re-annealed on a hot plate at 170°C for 3-5 minutes before any new measurements on the samples were undertaken to reconvert any newly-formed δ_H-phase (induced by the exposure to moisture) to the black α-phase and to ensure good film crystallinity.

2 X-ray diffraction

2.1 XRD spectra

The XRD patterns were measured in air using a Panalytical X'pert powder diffractometer with a copper X-ray source (Cu-Kα X-rays with a wavelength of 1.5418 Å).
The XRD patterns were then corrected for tilt by shifting the 2θ-axis, such that the z-cut quartz reference peaks at $2\theta = 16.433°$ were all aligned for the films on quartz substrates. The films deposited on sapphire substrates were shifted similarly to have their sapphire reference peak aligned at $2\theta = 20.553°$.

The extracted peak position and FWHM of each XRD peak that was studied were obtained manually using a smoothing algorithm to find the associated maximum, and the full widths at half maximum after the background were subtracted.

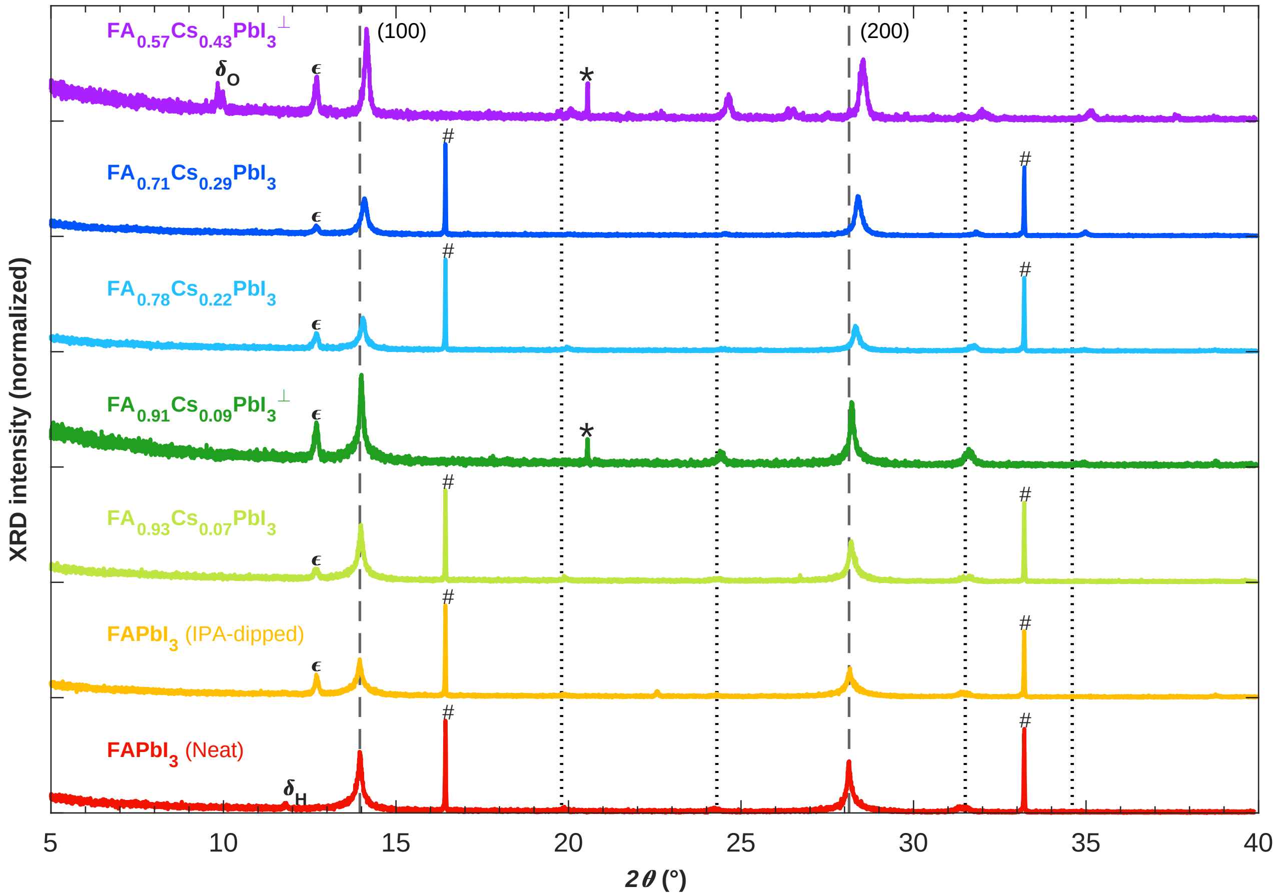

Supporting Figure S1: Full X-ray diffraction (XRD) pattern for the investigated $FA_{1-x}Cs_xPbI_3$ films. Substrate peaks are indexed with # and $*$ for quartz and sapphire substrates respectively. The main cubic (α) $FAPbI_3$ peaks ((100) and (200)) are represented by the vertical black dashed lines at the experimentally measured values of 14° and 28.2° respectively. The literature values[S2] of the less prominent cubic α-$FAPbI_3$ peaks are indicated by vertical black dotted lines, at 2θ values of 19.8°, 24.3°, 31.5°, and 34.6°. The PbI_2 peak can be seen for the films dipped in $CsCH_3COO$ IPA solution at 12.7°, and is indexed with ϵ.[S3] The most prominent XRD peak for the non-perovskite δ_H-$FAPbI_3$ and δ_O-$CsPbI_3$ phases appear for the $FAPbI_3$ neat film and the film with highest Cs percentage at 11.8°,[S2] and 9.9°,[S2–S4] respectively.

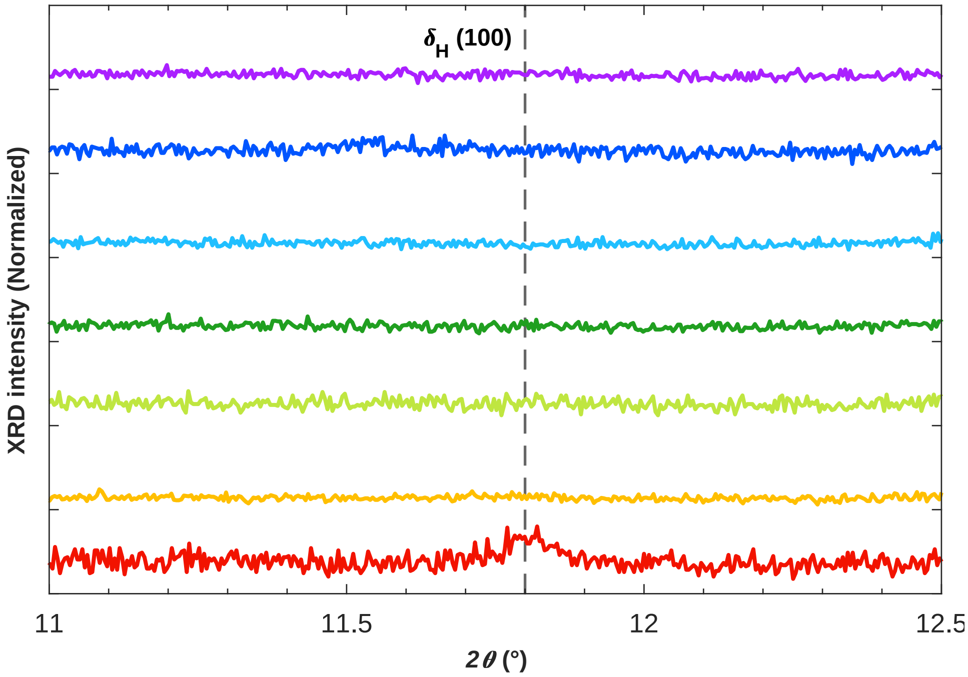

Supporting Figure S2: X-ray diffraction (XRD) pattern magnified around the hexagonal non-perovskite δ_H-$FAPbI_3$ phase for the investigated $FA_{1-x}Cs_xPbI_3$ films. The extracted position of the most prominent δ_H-$FAPbI_3$ peak (100)[S5,S6] is indicated by a vertical black dashed line. An observable peak is only found for the neat $FAPbI_3$ film. The labels for the different colored solid lines is included in Supporting Figure S1.

The increase in the FWHM of the XRD perovskite peaks with higher Cs-acetate concentrations (shown in Supporting Figure S3) is at odds with the improvement in crystallinity of the film and the enlargement of the grain size, which had previously been reported for the addition of Cs in the literature.[S7] This increase in FWHM, indicating a reduction in crystallographic purity, can be explained by the non-uniformity of the Cs cation exchange across the thickness of the film, and the formation of $CsPbI_3$,[S8] both of which will increase in prominence when a higher $CsCH_3COO$ dipping concentration is used.[S1] This conclusion is supported by the observation of a prominent non-perovskite δ_O-$CsPbI_3$ XRD peak for the highest Cs concentration film, presented in Supporting Figure S1.

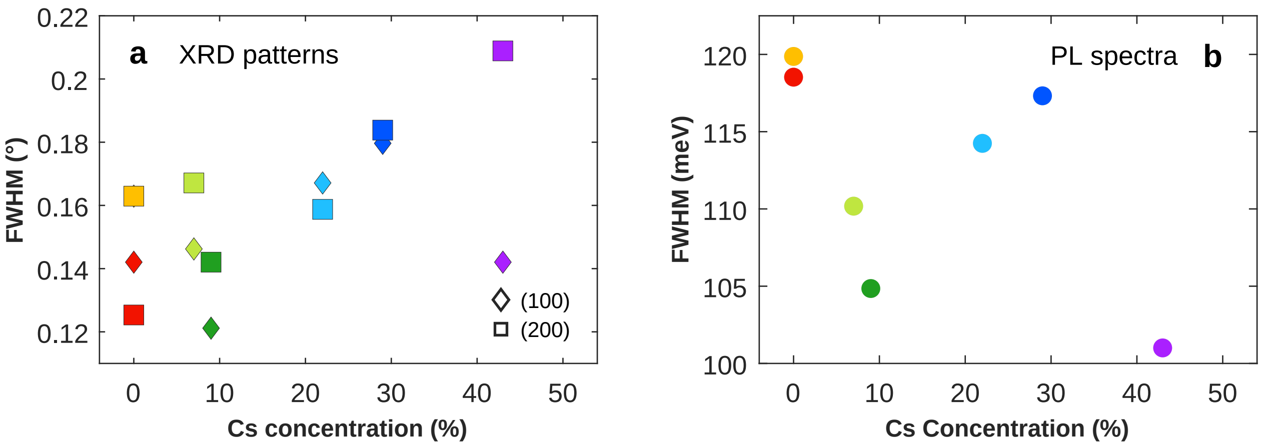

Supporting Figure S3: Full widths at half maximum (FWHM) of the prominent XRD perovskite peaks and the PL peaks for $FA_{1-x}Cs_xPbI_3$, as a function of Cs content. **a**: Extracted FWHM of the two most prominent XRD peaks (100), and (200) for the investigated films, represented with diamond and square markers respectively. **b**: Extracted FWHM of the main peak in the PL spectra.

2.2 Determining the Cs % of the films.

To determine the Cs percentages within the $FA_{1-x}Cs_xPbI_3$ films achieved through dipping in solutions of $CsCH_3COO$ of different concentrations in IPA, we pursued three different approaches which gave very similar results. In a previous study by Jiang et al.,[S1] which also introduced the fabrication procedure that we used, a linear relationship between the pseudo-cubic lattice parameter and Cs concentration within the film was extracted from the XRD patterns, X-ray fluorescence (XRF; which probes the bulk of the film) and X-ray photoelectron spectroscopy (XPS; which probes the surface of the film) measurements on similar films. Notably, the linear relationship between the lattice parameters and the Cs concentration obtained by Jiang et al. depends on the part of the film being probed. The procedure of partial substitution of FA by Cs implemented in both studies, results in a non-uniform, depth-dependent Cs concentration. In this situation, the surface ultimately has a higher concentration of Cs as a result of more exposure to the cation exchange-inducing solution. As a consequence, the Cs concentration on the surface - probed by XPS measurements, and in the bulk - probed by XRF measurements, yields different linear relationships between Cs percentages and pseudo-cubic lattice parameter. We hereafter use the bulk linear relationship as the relationship that best represents the majority of the film and the approximate mean Cs concentration present throughout the film. In the first two approaches, we therefore calibrated the lattice parameters of the films used in our study to the linear relationship experimentally obtained by Jiang et al.[S1]

In the first approach, we offset the measured XRD 2θ values of the films such that the (200) peak of the neat $FAPbI_3$ film measured in our study was aligned with the experimental (200) XRD peak of the $FAPbI_3$ film measured in the study by Jiang et al..[S1] Subsequently, their linear relationship was directly applied to our calculated lattice parameters to obtain approximate Cs concentrations (the results of this method are denoted “XRD offset”, and displayed in Supporting Figure S4a).

In the second approach, we attempted a physically more plausible method, in which the

XRD patterns were only shifted such that the reference quartz and sapphire peaks matched their literature values. In this method, we directly calculated the lattice parameters from the measured XRD patterns, and offset the linear relationship obtained from Jiang et al.,[S1] such that it would estimate zero incorporation of Cs into the lattice for the lattice parameter value of the neat $FAPbI_3$ film used in our study (the results of this method are denoted "Fit offset", and displayed in Supporting Figure S4b).

For the third approach, we implemented Vegard's law, which is a heuristic method that expects the pseudo-cubic lattice parameter of a solid solution to be the weighted mean of the lattice parameter of its constituents (the results of which are displayed in Supporting Figure S4c). The reference end points for this estimation were our measured pseudo-cubic lattice parameter for neat $FAPbI_3$ (of 6.339 Å), and the literature-extracted pseudo-cubic lattice parameter for $CsPbI_3$ (of 6.153 Å).[S1,S9,S10] The extracted Cs concentration from all three methods is reassuringly similar and predicts the Cs concentration range to roughly exist between 7%, and 43% Cs for the lowest (0.2 mg/mL) and highest (7.5 mg/mL) dipping concentration of $CsCH_3COO$ solutions in (see Supporting Figure S4d). Supporting Table S1 shows the measured 2θ values for the (200) XRD peak, the calculated pseudo-cubic lattice parameters and the estimated Cs concentration for each method. The calculated pseudo-cubic lattice parameter values change by around 1.4% between the neat $FAPbI_3$ film (0% Cs) and the $FA_{0.57}Cs_{0.43}PbI_3$ film (43% Cs).

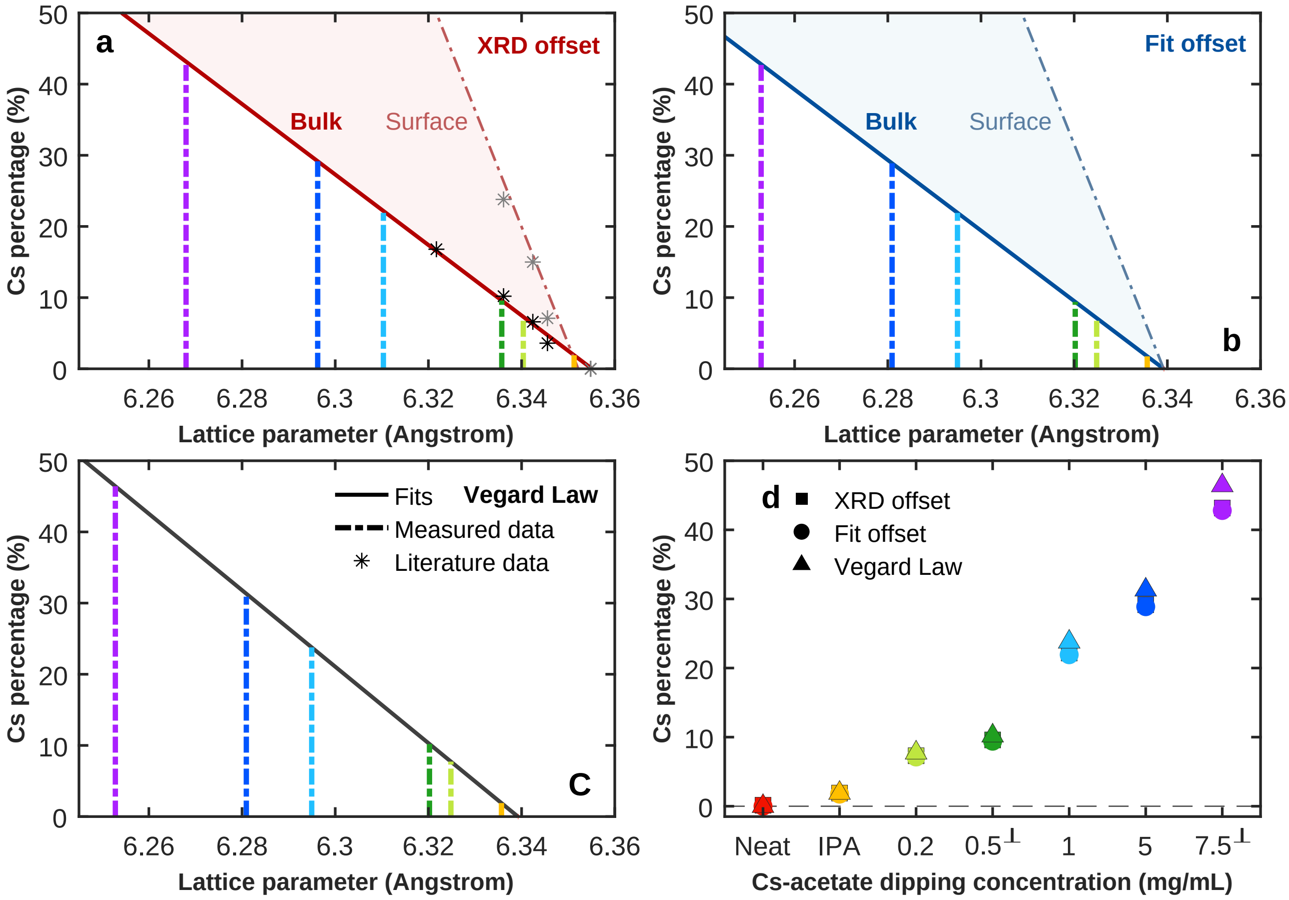

Supporting Figure S4: Extraction and calibration of Cs percentages in Cs-acetate dipped $FA_{1-x}Cs_xPbI_3$ films. (**a**) and (**b**) show the calibration method used to extract rough estimates for the Cs concentration of the films used in this study by either **a**: aligning the XRD patterns such that the pattern of the neat $FAPbI_3$ film matches that of the literature values, or **b**: offsetting the linear relationship between Cs concentration pseudo-cubic lattice parameter, to give the correct estimate for the $FAPbI_3$ film (0% Cs). The solid lines represent the extracted linear relationship between the pseudo-cubic lattice parameter and measured Cs content from Jiang et al.[S1] both for the bulk and the surface, while the dashed lines correspond to the lattice parameter values calculated for the films used in this study. (**c**) shows the Cs concentration estimates from Vegard's law. The reference end points for this heuristic estimation were our measured pseudo-cubic lattice parameter for neat $FAPbI_3$ (of 6.339 Å), and the literature-extracted pseudo-cubic lattice parameter for $CsPbI_3$ (of 6.153 Å).[S1,S9,S10] (The legend in (**c**) also applies to (**a**), and (**b**)). (**d**) shows the extracted Cs concentration from each of these different methods. (Films are evaporated on quartz or sapphire (indicated by $^{\perp}$) substrates).

Supporting Table S1: Parameters extracted from XRD patterns of $FA_{1-x}Cs_xPbI_3$ films. The (200) XRD 2θ peak position and corresponding calculated pseudo-cubic lattice parameter for films dipped in IPA solutions with differing Cs-acetate concentrations. The Cs concentration is extracted using either the XRD offset method, Fit offset method, or Vegard's law - as explained and discussed in SI Section 2. (Films are evaporated on either quartz or sapphire (indicated by $^{\perp}$) substrates).

**Cs-acetate concentration**	**XRD (200)**	**Lattice parameter**	**Cs XRD Offset**	**Cs Fit Offset**	**Cs Vegard**
Neat	28.13°	6.339 Å	0%	**0%**	0%
IPA-dipped	28.15°	6.336 Å	0%	**0%**	0%
0.2 mg/mL	28.20°	6.325 Å	7.3%	**7.1%**	7.7%
0.5 mg/mL$^{\perp}$	28.22°	6.320 Å	9.6%	**9.4%**	10.2%
1 mg/mL	28.33°	6.295 Å	22.2%	**21.9%**	23.8%
5 mg/mL	28.40°	6.281 Å	29.2%	**28.9%**	31.3%
7.5 mg/mL$^{\perp}$	28.53°	6.253 Å	43.2%	**42.8%**	46.4%

3 Steady-state photoluminescence

A 398 nm diode laser (PicoHarp, LDH-D-C-405M) was used to photo-excite the samples, on a continuous wave setting at an intensity of 75.5 mW/cm^2. The resultant PL was collected and coupled into a grating spectrometer (Princeton Instruments, SP-2558), which directed the spectrally dispersed PL onto a silicon iCCD (intensified charge coupled device, PI-MAX4, Princeton Instruments). The samples were mounted in a vacuum cell under low pressure ($\sim 10^{-2}$ mbar).

The PL peak position and full width at half maximum (FWHM) were obtained manually using a smoothing algorithm to smooth the measured data and find the maximum, and the full width at half maximum after the background had been subtracted. The PL amplitude is seen to decrease for the films with increasing Cs content, suggesting an increase in the trap density within the film and an increased prevalence of nonradiative monomolecular charge-carrier recombination. This could be explained by the fabrication method used in this study: the composition differential throughout the thickness of the film for higher Cs films could create a tail of deep trap states, and the solution-induced cation exchange could also be introducing Cs interstitials and leading to the creation of more trap states in the film.

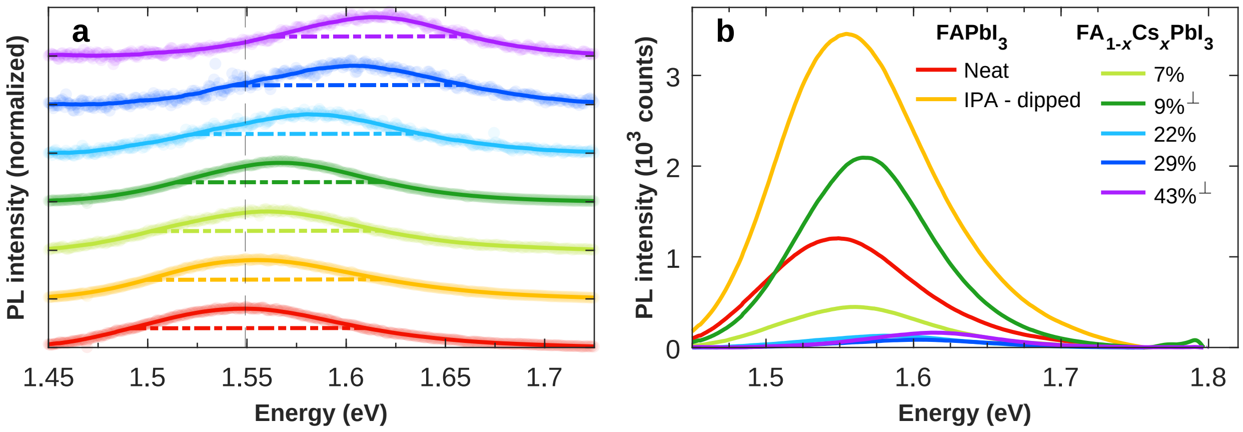

Supporting Figure S5: Photoluminescence spectra of the $FA_{1-x}Cs_xPbI_3$ films. **a**: Normalized PL spectra (scatter points) for the different films, with smoothed versions of the spectra shown as solid lines. For visual clarity, the spectra are vertically offset, the full-width-half-maximum (FWHM) is represented by the horizontal dashed lines, and the PL peak of the neat film is represented by the vertical dashed line. (**b**) shows the unnormalized, smoothed PL spectra. The PL amplitude is observed to decrease for the films with increasing Cs content. (The legend in (**b**) also applies to (**a**). Films are evaporated on quartz or sapphire (indicated by $^\perp$) substrates).

4 Absorption measurements

Reflectance (R) and transmittance (T) spectra were measured using a Fourier transform infrared (FTIR) spectrometer (Bruker Vertex 80v), configured with a tungsten halogen lamp illumination source, a CaF_2 beamsplitter and a silicon detector. The absorption coefficient (α) spectrum of each sample can then be obtained using the relationship

$$\alpha = -\frac{1}{d} \cdot \log_{10}\left(\frac{T}{1-R}\right) \tag{S1}$$

where d is the film thickness (100 nm). The measured transmittance and reflectance spectra of the $FAPbI_3$ (Neat) film at 4 K, along with the calculated absorption coefficient spectra is displayed in Supporting Figure S6.

The samples were all mounted in a gas-exchange helium cryostat (Oxford Instruments, OptistatCF2) in a helium atmosphere for the room-temperature measurements (where the outer vacuum chamber was pumped down to low pressures ($< 5 \times 10^{-5}$ mbar)) and for the temperature-dependent study, where the temperature was varied between 4K and 295K in either 5K or 10K increments.

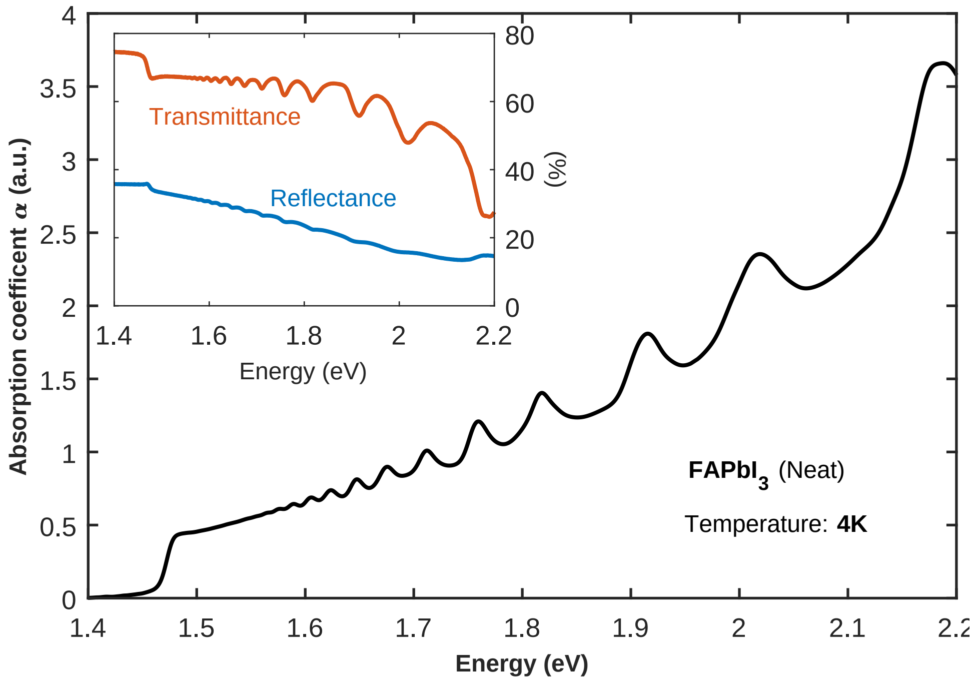

Supporting Figure S6: Absorption coefficient spectra of the $FAPbI_3$ (Neat) film. Absorption coefficient spectrum of the $FAPbI_3$ (Neat) film at 4 K, calculated from the measured transmittance and reflectance spectra shown in the inset.

4.1 Elliott fitting

The onset in the absorption coefficient spectrum was fitted using Elliott's model that accurately describes the absorption of a semiconductor near its band edge.[S11]

The theory expresses the energy-dependent absorption as:

$$\alpha(E) = \alpha_{\mathrm{X}}(E) + \alpha_{\mathrm{C}}(E) \tag{S2}$$

The bound exciton contribution α_{X} has the form:

$$\alpha_{\mathrm{X}}(E) = \frac{b_0}{E}\sum_{n=1}^{\infty}\frac{4\pi E_{\mathrm{B}}^{3/2}}{n^3}\delta\left(E - \left[E_{\mathrm{g}} - \frac{E_{\mathrm{B}}}{n^2}\right]\right) \tag{S3}$$

where b_0 is a constant of proportionality that incorporates the electric dipole transition matrix element between the valence and conduction band. α_{X} is formed of the weighted sum of contributions from the exciton states with positive integer quantum number n and energies $E_{\mathrm{g}} - \frac{E_{\mathrm{B}}}{n^2}$, where E_{g} is the bandgap energy and E_{B} is the exciton binding energy.

Here, the contribution from the electron-hole continuum states $\alpha_{\mathrm{C}}(E)$ has the form

$$\alpha_{\mathrm{C}}(E) = \frac{b_0}{E}\left[\frac{2\pi\sqrt{\frac{E_{\mathrm{B}}}{E-E_{\mathrm{g}}}}}{1-\exp(-2\pi\sqrt{\frac{E_{\mathrm{B}}}{E-E_{\mathrm{g}}}})}\right]c_0^{-1}\mathrm{JDoS}(E) \tag{S4}$$

where the joint density of states is given by $\mathrm{JDoS}(E) = c_0\sqrt{E-E_{\mathrm{g}}}$ for $E > E_{\mathrm{g}}$ and 0 otherwise, and the joint density of states constant $c_0 = \frac{1}{(2\pi)^2}\left(\frac{2\mu}{\hbar^2}\right)^{3/2} \times 2$, where μ is the reduced effective mass of the electron-hole system. The term in square brackets is the Coloumbic enhancement factor, which increases the absorption from the continuum states above the square-root form of the JDoS for a direct-gap semiconductor as a result of the Coloumbic attraction between the unbound electrons and holes.

The linear combination of the contributions from the excitonic and continuum states, $\alpha(E)$,

is then convolved with a normal distribution - representing the homogeneous broadening caused by electron-phonon coupling. The broadening is mathematically denoted by $g(E) = \mathcal{N}(0, \gamma^2) = \frac{1}{\sqrt{2\pi}\gamma} e^{-\frac{x^2}{2\gamma^2}}$.

The bandgap energy (E_g), amplitude (Amp), and electron-phonon coupling strength (γ) are freely fitted to yield the values shown in Supporting Table S2. The exciton binding energy (E_B) was globally fixed to 2 meV, based on $FAPbI_3$ room-temperature values (calculated from Elliott fits to the absorption onsets) from the literature,[S12,S13] and the reported experimental invariance of E_B with the $FA_{1-x}Cs_x$ A-cation ratio (measured from magneto optical studies) at lower temperatures.[S14,S15]

Supporting Table S2: Parameters extracted from Elliott fits to the absorption onsets of $FA_{1-x}Cs_xPbI_3$ films. The absorption coefficient spectra were fitted with 3 free parameters: E_g representing the optical bandgap, γ representing the electron-phonon coupling, and Amp representing the amplitude of the spectrum. The exciton binding energy E_B was globally fixed to a value of 2 meV.[S12–S15]

Cs concentration	E_g (eV)	E_B (meV)	γ (meV)	Amp (a.u.)
0% (Neat)	1.554	2	32.3	18.6
0% (IPA-dipped)	1.558	2	33.9	15.5
7%	1.564	2	31.5	19.6
9%	1.568	2	34.4	23.3
21%	1.590	2	32.2	27.1
29%	1.601	2	30.4	27.2
43%	1.612	2	43.1	34.0

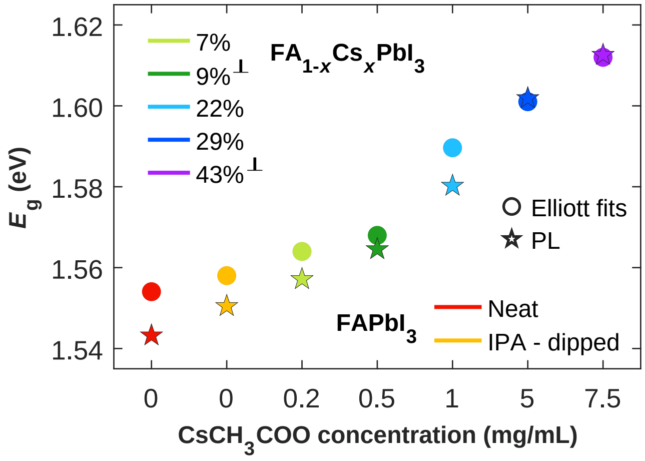

Supporting Figure S7: Optical bandgap of $FA_{1-x}Cs_xPbI_3$ films. The optical bandgap for all $FA_{1-x}Cs_xPbI_3$ films extracted either from room-temperature steady-state PL spectra (star markers), or from the Elliott fits to the absorbance onset (circle markers). The Stokes shift is the difference between the absorbance-extracted bandgap and the PL-extracted bandgap. The small difference between the absorbance and PL bandgaps also suggests limited photon recycling and reabsorption events, which would otherwise cause an observable red-shift of the PL spectrum and peak.

4.2 Tauc plots

Tauc plots are frequently used in the literature to extract the bandgap of perovskite polycrystalline thin films including $FA_{1-x}Cs_xPbI_3$ films,[S8,S16] yet their validity is somewhat questionable. The Tauc relationship was derived for amorphous semiconductors and its apparent close proximity to the actual perovskite bandgap is dependent on some of the errors from its assumptions cancelling out each other.[S17]

Despite its deficiencies, we here apply this relationship to our data in order to make a comparison with a literature value of the bandgap of a similar material,[S18] which was however obtained using Tauc plots and an integrating sphere to account for scattered light. The extracted bandgaps we obtain from Tauc plots to the absorption onsets of $FA_{1-x}Cs_xPbI_3$ are noticeably smaller than the optical bandgaps which were extracted from PL and Elliott fits, which would indeed imply an unlikely negative Stokes shift. However, they generally follow the same trend of a widening bandgap for the films dipped in the higher $CsCH_3COO$ concentration solutions.

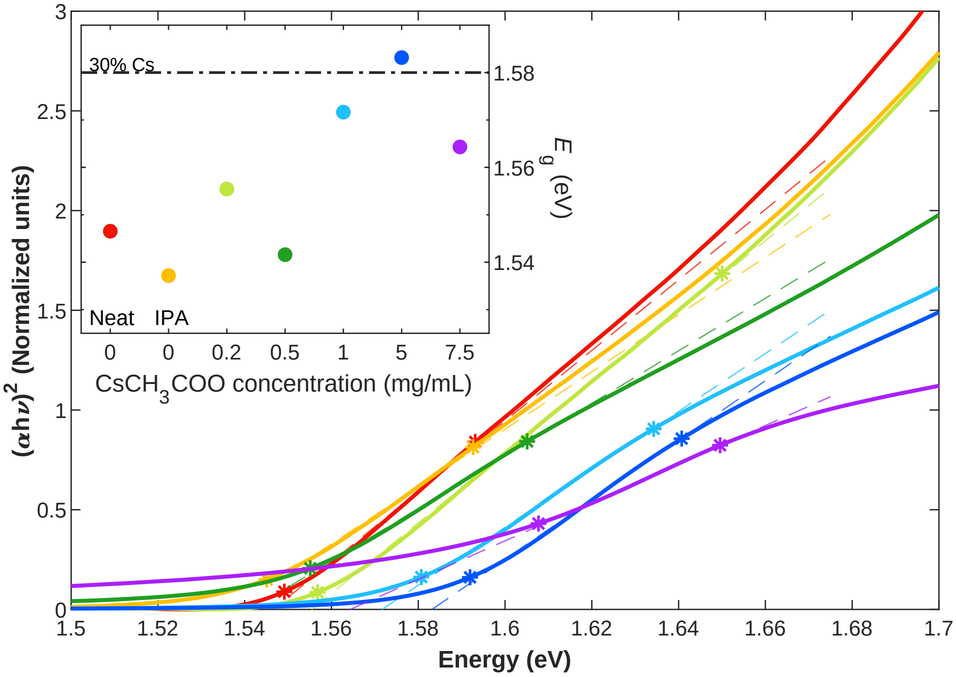

Supporting Figure S8: Tauc plots to onsets of the squared absorbance spectra of $FA_{1-x}Cs_xPbI_3$ films. $(\alpha h\nu)^2$ (solid lines), and the linear fits (dashed lines) used for bandgap extraction for direct bandgap semiconductors according to the Tauc method. The inset shows the variation of the Tauc-extracted bandgaps, E_g, across the different Cs concentrations used for dipping $FAPbI_3$ films and a literature Tauc-extracted bandgap for an $FA_{0.7}Cs_{0.3}PbI_3$ film shown by the black dot-dash line.[S18]

4.3 Baseline fitting and peak indexing

The Elliott fitting is only applicable to the absorption onset near the band edge, where the electronic band structure can be assumed to be parabolic and isotropic, transitions can be assumed to be between one valence and one conduction band, and the electric dipole transition matrix assumed to be independent of photon energy.[S19] Therefore to decouple the peak features from the standard absorption coefficient spectrum at energies higher than the bandgap, we used a phenomenological fit to extract the peaks and quantify their behaviour.

The positions of the troughs in the experimental absorption coefficient data were obtained by subtracting a smoothed "central fit" line from the measured spectrum to result in a spectrum showing only the peak feature, from which the minimas (corresponding to the troughs) could be more readily extracted. Subsequently, interpolating a cubic spline between the experimental data at the energies of these troughs, resulted in the spline baseline fit we used to decouple the peak features from the underlying absorption coefficient data.

It is worth noting that because the dimensionality of confinement has not been conclusively determined, the peak-decoupling procedure implemented in this study potentially eliminates any underlying density of state contributions that may be associated with the quantum confinement. For instance, for an infinite-quantum-well confinement in all three dimensions, the density of states will have peak-like behaviour similar to what we extract from the decoupling mechanism. Alternatively, for a one-dimensional or a two-dimensional electronic confinement, the density of states will have additional continuum states associated with the electronic free motion in the other non-confined dimensions, states that would be removed when using this decoupling mechanism and would result in the same peak-like features we observe. This makes an accurate estimate of the confinement dimensionality even more difficult.

In the quantitative analysis of the quantum confinement and behaviour of the peaks implemented in this study, we utilise the confinement energy and spectral area under the

peaks. We define the confinement energy of a peak to be the Elliott-extracted bandgap of the material subtracted from the energy of the decoupled peak. The indexing of the peaks is such that peak #0 is the lowest energy discernible peak for a $FAPbI_3$ film at 4 K,[S13] in a consistent manner with our previous indexing method. Accordingly, the highest-energy discernible peaks of all the films have the same highest peak index. Meanwhile, the percentage spectral area under the peaks is defined as the percentage of the spectral area between the experimental absorption coefficient data and baseline fit (are highlighted in pink in Supporting Figure S9) from the total experimental spectral area (sum of the areas highlighted in pink and grey in Supporting Figure S9) (between the first and last detectable minima of the peak features). The definitions of the nomenclature and indexing used in this study, are visually represented in Supporting Figure S9

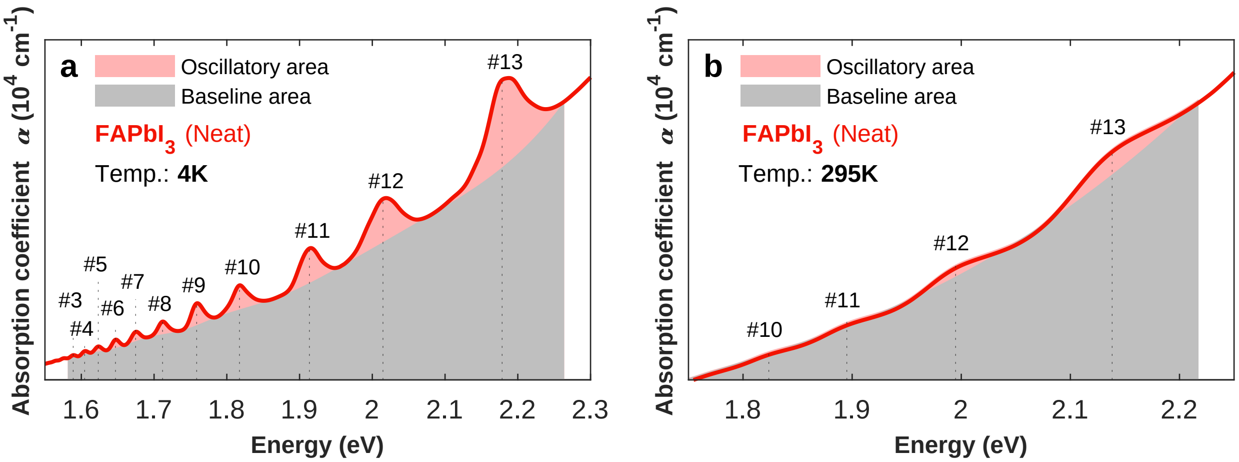

Supporting Figure S9: Absorption coefficient spectrum of $FAPbI_3$ with definitions of terms used in the analysis. Absorption coefficient spectrum of the neat $FAPbI_3$ film, **a**: at 4K, and **b**: at room temperature. The spectral area under the spline baseline (grey-shaded area) and spectral area between the measured absorption coefficient and spline baseline (pink-shaded area) - termed the peak area, used to calculate the percentage of spectral area under the peaks in Fig 3a, and Fig 3a, are visually represented. The calculated spectral area under the peaks is calcualted The relevance of the spectral area under the peaks compared to the peak amplitude, is that it takes into account both the extracted amplitude and FWHM of the peaks, making it comparatively less susceptible to artificial changes caused by the narrowing and widening of the peaks as opposed to changes caused by the increase and decrease in strength of confinement. The peaks in Fig 3c, and Fig 3d are identified by their peak index, with the lowest energy discernible peak labelled as peak #0.

4.4 Temperature-dependent study of the absorbance spectra

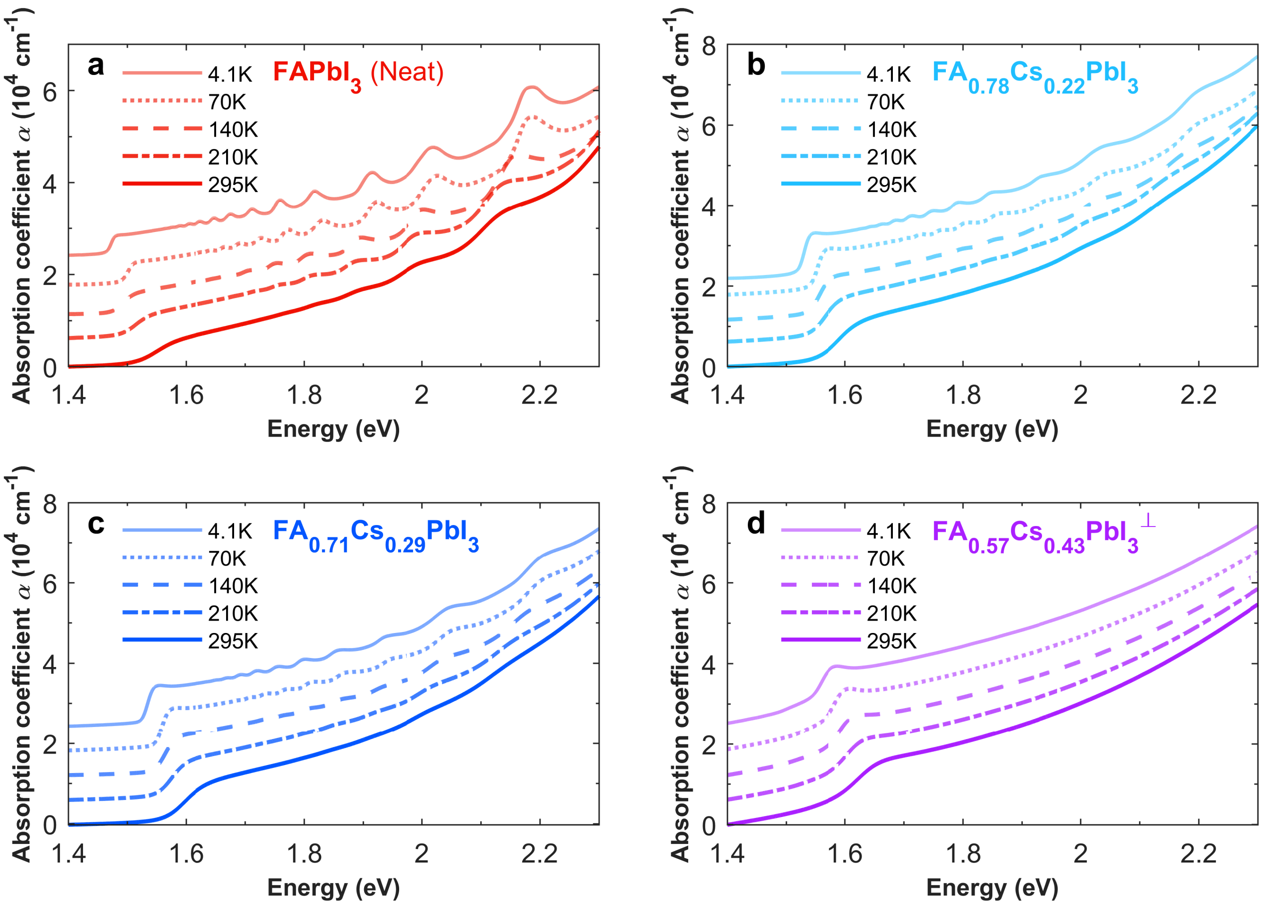

Supporting Figure S10: Temperature-dependent absorption coefficient spectra of $FA_{1-x}Cs_xPbI_3$. **a**: $FAPbI_3$ (Neat), **b**: $FA_{0.78}Cs_{0.22}PbI_3$, **c**: $FA_{0.71}Cs_{0.29}PbI_3$, **d**: $FA_{0.57}Cs_{0.43}PbI_3$. Films are evaporated on either quartz or sapphire (indicated by $^{\perp}$) substrates.

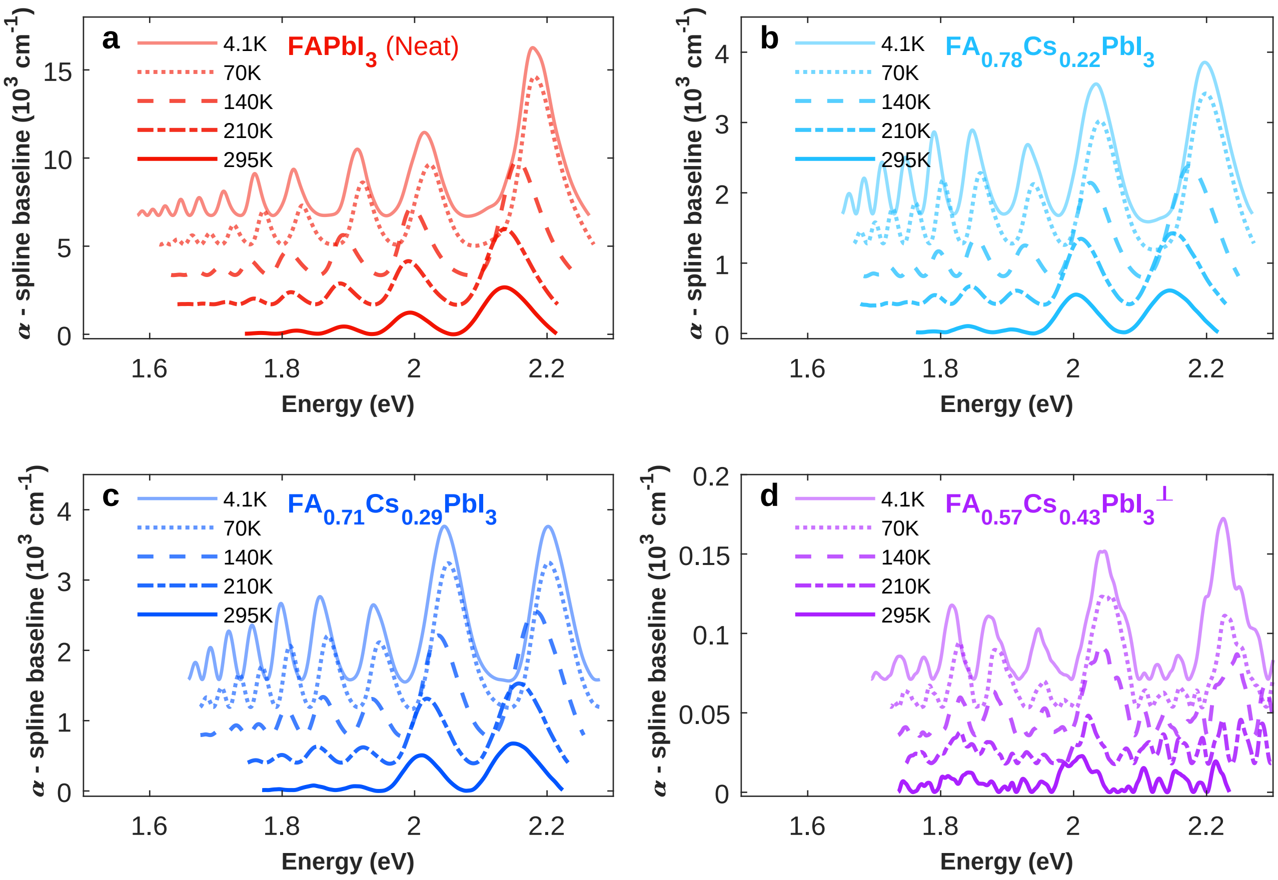

Supporting Figure S11: Temperature-dependent above-bandgap peaks features of $FA_{1-x}Cs_xPbI_3$. The peaks are decoupled from the absorption coefficient spectra in the same way as described in the main text. **a**: $FAPbI_3$ (Neat), **b**: $FA_{0.78}Cs_{0.22}PbI_3$, **c**: $FA_{0.71}Cs_{0.29}PbI_3$, **d**: $FA_{0.57}Cs_{0.43}PbI_3$. Films are evaporated on either quartz or sapphire (indicated by $^{\perp}$) substrates.

5 Theoretical models

5.1 Infinite quantum well

The infinite quantum well provides an easy and straightforward method, albeit only an approximation to the real scenario, to analyse the peak behaviour and obtain simple analytic solutions that can be a good approximation to the deep states within the quantum well. Assuming a perfectly parabolic electronic density of states for a confined system in an infinite quantum well, the confinement energy of the nth peak is given by

$$E_n = \frac{\hbar^2}{8\mu^* L^2} \sum_{i=1}^{d_{\mathrm{conf}}} n_i^2 \tag{S5}$$

where μ^* is the reduced effective mass of the electron and hole in the material - defined as $\frac{1}{\mu^*} = \frac{1}{m_e^*} + \frac{1}{m_h^*}$, L is the quantum well width, and d_{conf} is the dimensionality of confinement.

We fit the 4 K confinement energies extracted from the absorbance spectra against their peak indices with a one-dimensional infinite quantum well model (shown in SI Figure S12) given by

$$E_n = A(n - n_0)^2 + B \tag{S6}$$

where A is a constant of proportionality $\left(\frac{\hbar^2}{8\mu^* L^2}\right)$, n_0 is the peak index at which the quadratic relation starts (lowest discernible peak $n_0 = 0$ for the neat $FAPbI_3$ film at 4 K), and B is the energy shift of the confined ground state from the bulk bandgap, which could be a result of a different bandgap within the confined regions owing to the lattice strain, and was fixed to the freely fitted value obtained from the $FAPbI_3$ fit across the different samples.

The factor A changes by 9.6% between the neat $FAPbI_3$ film (0% Cs) and the $FA_{0.71}Cs_{0.29}PbI_3$ film (29% Cs), which is roughly consistent with what is observed for the global linear regression for the confinement energies at room temperature across the different $FA_{1-x}Cs_xPbI_3$ films.

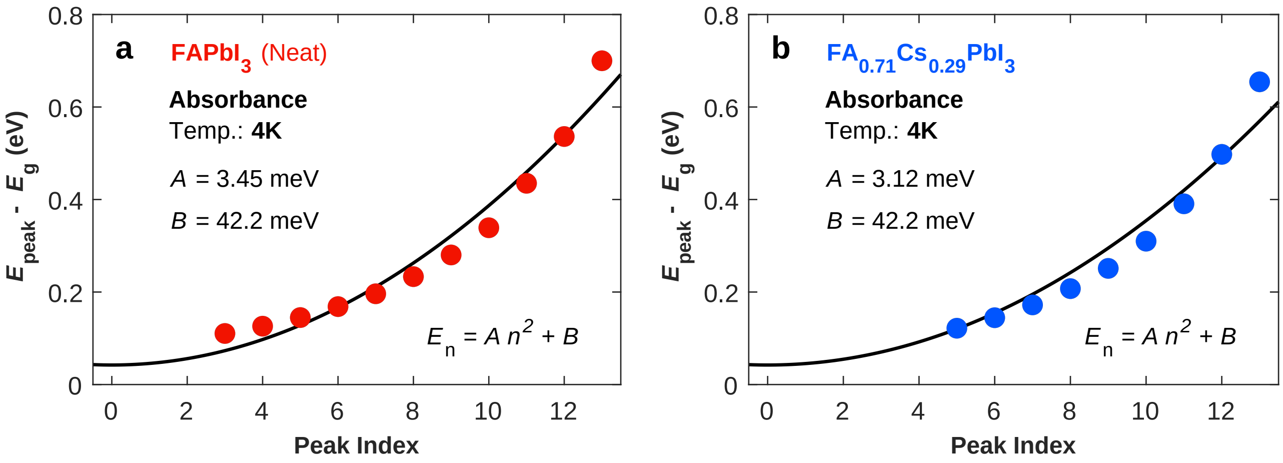

Supporting Figure S12: Quadratic confinement models for the infinite potential well using the absorbance peak features for $FA_{1-x}Cs_xPbI_3$ films. The confinement energies from the absorbance spectra of **a**: $FAPbI_3$ (Neat) film, and **b**: $FA_{0.71}Cs_{0.29}PbI_3$ film are fitted with a quadratic model according to the infinite quantum well model.

5.2 Krönig-Penney potential

The Krönig-Penney model offers a more realistic model to be used, in which the quantum confinement is the result of a periodic potential barrier of finite height, perhaps originating from periodic δ_H-$FAPbI_3$ phase inclusions for lattice strain relaxation. The simple Krönig-Penney approach can be applied here to a superlattice of periodically arranged finite quantum wells and has been shown to yield similar results to those obtained from a infinite quantum well for modelling the quantum confinement being investigated in this study.[S13]

6 Temperature-dependent study of steady-state PL

6.1 IPA-dipped $FAPbI_3$

Photon reabsorption and recycling events:

The overlaid absorbance and PL spectra for the IPA-dipped $FAPbI_3$ film (displayed in Supporting Figure S13) highlight the limited photon reabsorption occurring within the film, as will be discussed in this section. We note that peak features extracted from both optical characterization techniques occupy the same energy range (as can be seen in Fig 4), and yet they are slightly out of phase with respect to each other.

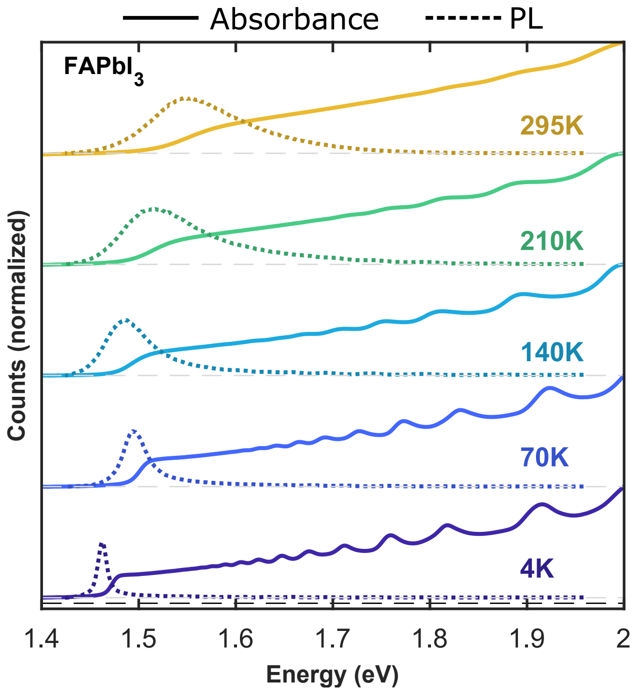

Supporting Figure S13: Photoluminescence and absorbance spectra of $FAPbI_3$ at different temperatures. Solid lines represent the absorption coefficient spectra, and dashed lines represent the photoluminescence spectra.

To conclusively refute the plausibility of photon recycling and reabsorption events modulating the PL spectra and artificially generating the PL peak features, we point out three main arguments. First, the spectra are thermally broadened because the distribution function governing the occupation of states near the band edge will tend to a Maxwell-Boltzmann thermal distribution, which would then be truncated on the high-energy end if photon absorption were prevalent. However, as the overlaid spectra in SI Figure S13 showcase, the PL spectra still possess a clear high-energy tail that extends well into the highly-absorbing regions in the absorbance spectrum. This is unlike what would be expected in the case that photon reabsorption and recycling events were prevalent, in which case the PL spectra would have been red-shifted beyond the absorbance onset and an asymmetric PL spectrum with a low-energy tail would have artificially emerged. Second, the very small film thickness - of around 100 nm, limits the amount of photon recycling occurring in the films and the degree to which the PL can be modulated by the absorbance spectrum. The penetration depth for the IPA-dipped $FAPbI_3$ film starts to decline below 100 nm for energies above 2.7 eV, highlighting the complete absorption of the excitation laser and the inability of the film to efficiently modulate the PL being emitted. Specifically, the penetration depth for the energetic range associated with the absorbance and PL features investigated in this study (between 1.55 eV and 2.25 eV) ranges from 3.2 μm to 220 nm.

Finally, we simulate the hypothetical shape of an originally smooth PL spectrum that has been filtered by (i.e. modulated by the absorption spectrum of) the entire IPA-dipped $FAPbI_3$ film. We then compare this spectrum with what we observed experimentally. We considered two possible baselines to represent the smooth PL emission spectrum: a smoothed baseline and a baseline that goes through the most prominent PL peak features as the hypothetical high-energy PL tails (see SI Figure S14a). By multiplying these hypothetical tails with the transmittance function of the entire film (displayed in SI Figure S14b), we obtained the filtered PL that would have been emitted if no PL peak features were present (see SI Figure S14c). A couple of observations can be made from this attempted imitation. First, the

generated PL tail has significantly less prominent peak features, even though this model is clearly the most extreme case, in which all the PL is assumed to pass through the entire film before collection. This highlights the need for substantially stronger and more prominent transmittance features to exist for PL peak features similar to what is observed to emerge - even for a model that over-exaggerates the effects of photon reabsorption. Second and more importantly, the amplitude of the absorbance features is highest for higher energies, which would naturally cause a similar amplitude variation with energy for the PL features if they indeed originated from photon recycling and absorbance modulations of the PL spectra as seen for the artificially generated PL peak features (displayed in SI Figure S14d). However, we observe the opposite amplitude dependence with energy for the actually observed PL peak features, in which the lower-energy confined states seem to have the largest PL amplitude. This amplitude variation can instead be understood in terms of charge-carrier relaxation, where charge carriers from the higher-energy confined states relax into the lower-energy confined states at a rate slightly faster than other de-excitation pathways, including funnelling to bulk regions, explaining the higher intensity at lower energies.

We note that the energetic difference between the PL and absorbance features can be better understood in terms of a combination of some Stokes shift, charge-carrier migration to domains with weaker confinement, or some weak influence of the absorption spectrum from photon reabsorption and recycling. The quantum confinement will create different electronic bands, and the relaxation of the photogenerated charge-carriers in each band to their local minima will define each band's Stokes shift. That Stokes shift will naturally be very different to the shift observed for the main PL peak, owing to the differing curvature and band structure between the band edge and higher up in the band.

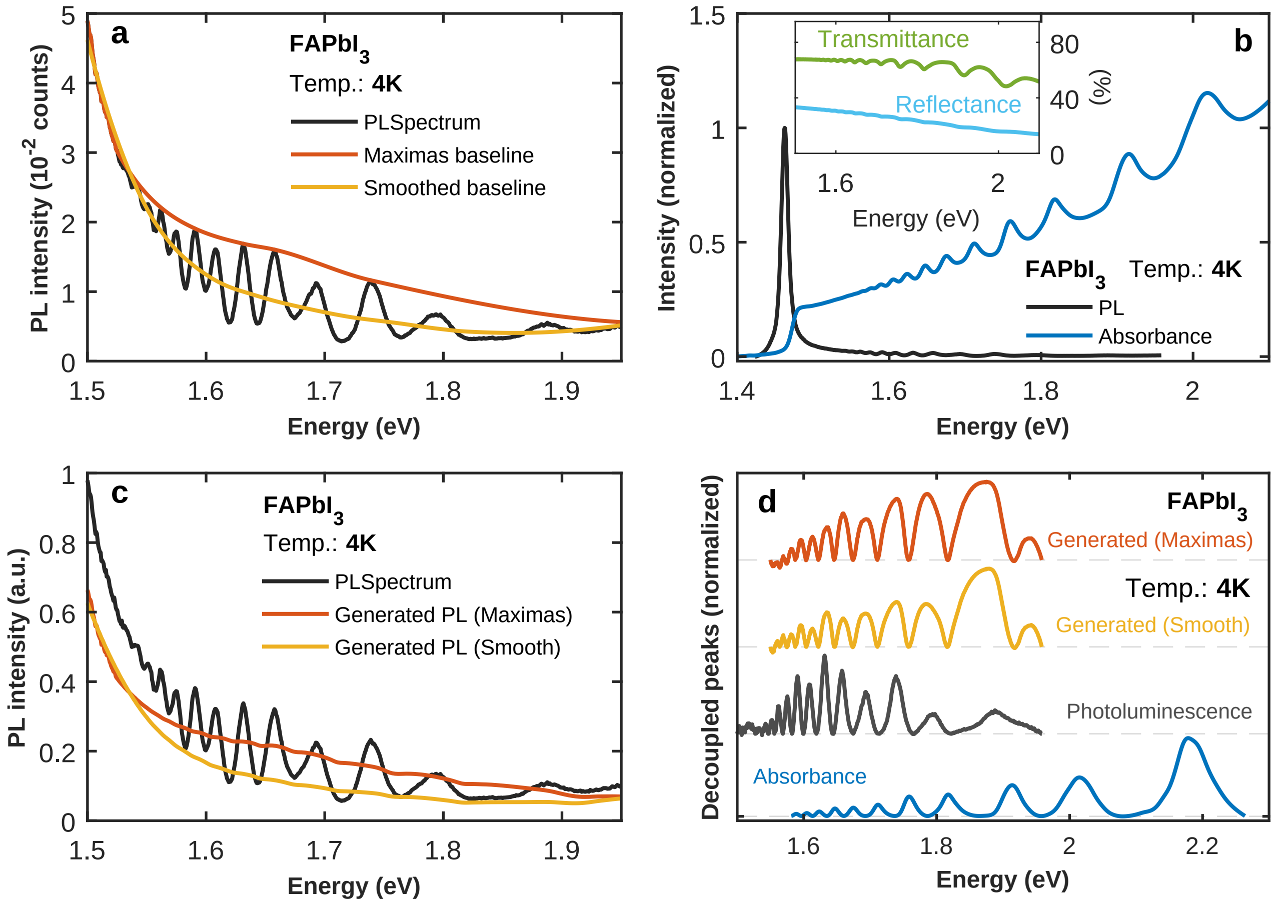

Supporting Figure S14: Quantitative imitation of the PL peak features observed for IPA-dipped $FAPbI_3$ at 4 K. (**a**) shows the high-energy tail of the 4 K PL spectrum of an IPA-dipped $FAPbI_3$ film with the noticeable PL peak features, along with the hypothetical tails for when no PL peak features would be present. (**b**) shows the full PL, absorption coefficient, reflectance, and transmittance spectra at 4 K. (**c**) shows the generated PL spectra ($\times$250) at 4 K for when the entire $FAPbI_3$ film is used as a filter for a hypothetical smooth PL without any peak features using the two baselines visualized in (**a**). (**d**) shows the decoupled peak features from the experimentally measured absorbance and Pl spectra, as well as the generated PL spectra in (**c**).

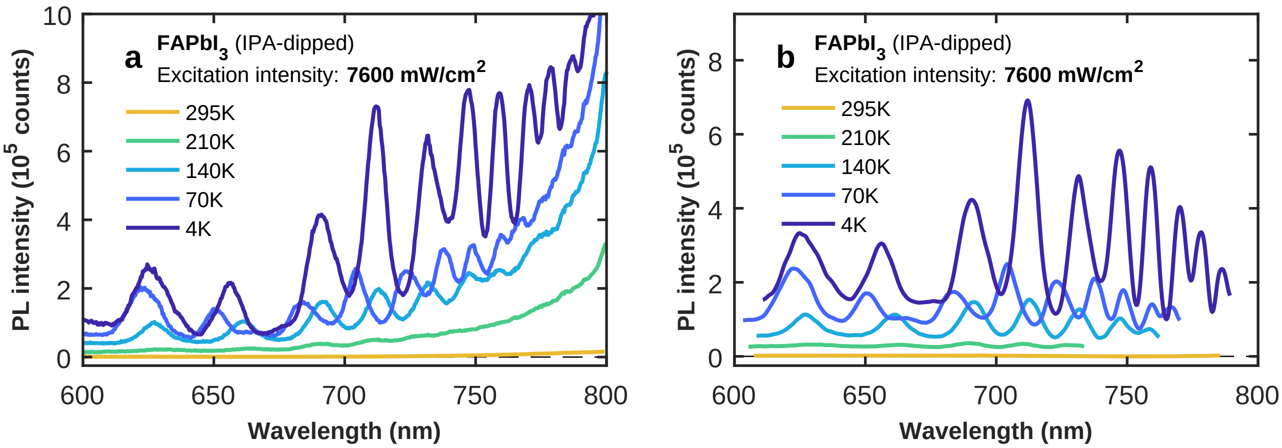

Supporting Figure S15: Photoluminescence spectra of the PL peak features in $FAPbI_3$ (IPA-dipped) at energies above the main band edge peak at different temperatures. (**a**) shows the absolute PL spectra of the IPA-dipped $FAPbI_3$ film at different temperatures for a laser excitation intensity of 7600 mW/cm^2. (**b**) shows the absolute peaks, decoupled from the PL spectra using a spline baseline fitting, for a laser excitation intensity of 7600 mW/cm^2, with subsequent curves vertically offset by 0.25×10^5 counts.

We further note that the spectrum of the decoupled PL peaks does not change much with a variation in the excitation intensity of the 398nm laser (as can be seen in Supporting Figure S16). This is further discussed in Section 6.1.1 in the SI.

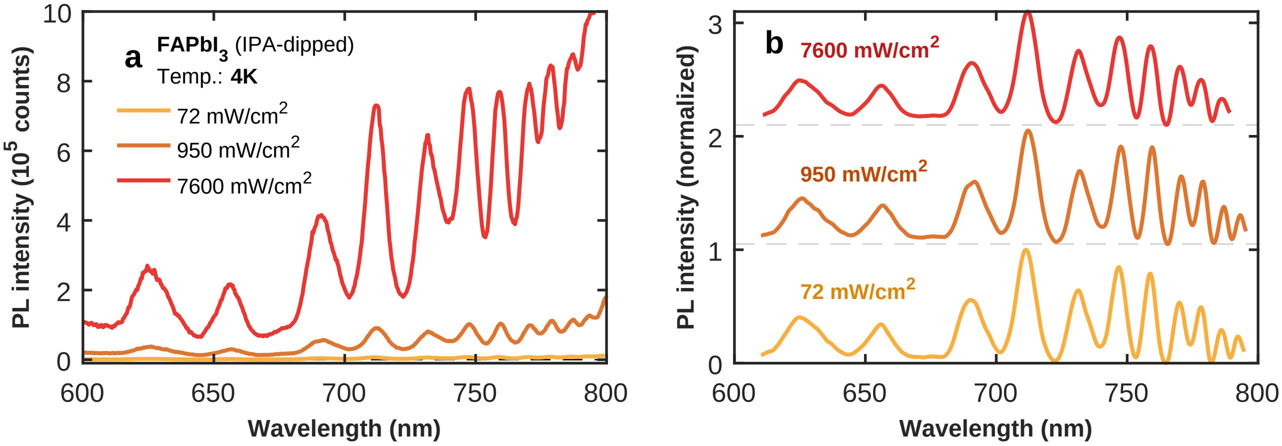

Supporting Figure S16: Photoluminescence spectra of the PL peak features in $FAPbI_3$ (IPA-dipped) at energies above the main band edge peak under different excitation intensities. (**a**) shows the absolute PL spectra of the IPA-dipped $FAPbI_3$ film at 4 Kelvin under difference excitation intensities. (**b**) shows the normalized peaks, decoupled from the PL spectra using a spline baseline fitting, under the different excitation intensities, with subsequent curves vertically offset for visual clarity.

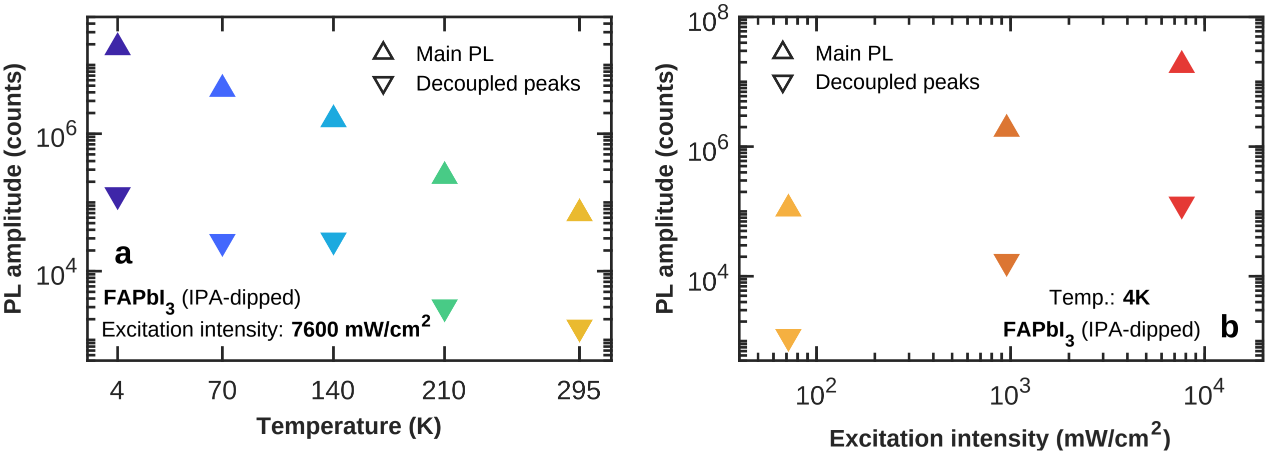

Supporting Figure S17: PL amplitudes for $FAPbI_3$ films as a function of temperature and excitation intensity. **a**: The temperature dependence and **b**: excitation intensity dependence of the extracted absolute amplitude of the main steady-state PL peak and that of the decoupled peaks is displayed. The amplitude of the decoupled peaks increases for both a reduction in temperature and a rise in excitation intensity, but to a lesser extent when compared to the main PL amplitude variation with temperature and excitation intensity (i.e. the relative amplitude of the decoupled peak features to the main PL decreases for both a reduction in temperature or a rise in excitation intensity.)

6.1.1 Confinement energy

A couple of things are worth noting regarding the observed PL peak features. First, we note that according to our primary model, these emissions originate from transitions between confined levels. Such emissions from higher-lying bands have however only been observed from zero-dimensional quantum dots (according to our literature search).[S20–S25] Due to the very fast relaxation to the lower-lying confined bands expected for such confined systems, these emissions typically have a strong dependence on excitation intensity, as they ultimately emerge from the slowing down of the relaxation dynamics due to either ground-state filling or hot phonon bottlenecks.[S25,S26] The spectra of the decoupled PL peaks of IPA-dipped $FAbI_3$ at 4 K (as observed in SI Figure S16) do not show any obvious dependence on excitation intensity - ruling out ground-state filling as a cause of these emissions. We instead argue that these emissions are observed as a snap-shot of early times after photoexcitation, when charge carriers experience competing fast radiative recombination, funnelling out of the confined domains to the surrounding bulk phase material, and internal relaxation within the confined bands. This can be due to some possible hindrance from complex selection rules and some moderate phonon bottleneck - which has been observed in perovskite 2D material before,[S27] and is possibly enhanced because of lower temperatures. Second, the relationship between the PL confinement energies (PL peak positions relative to the PL optical bandgap) against their peak index at 4 K can be modelled and fitted with a quadratic fit (shown in SI equation S6). In fact, this quadratic function fitted the PL confinement energies particularly well and yielded fitting parameters of the same order of magnitude as those obtained from the fitting of the absorbance confinement energies in SI Section 5 (displayed in Supporting Figure S18b). This agreement with the infinite quantum well model further corroborates the existence of light-emission from intrinsically quantum-confined states (albeit the fitted parameters are slightly different between the fits from the absorbance and PL confinement energies).

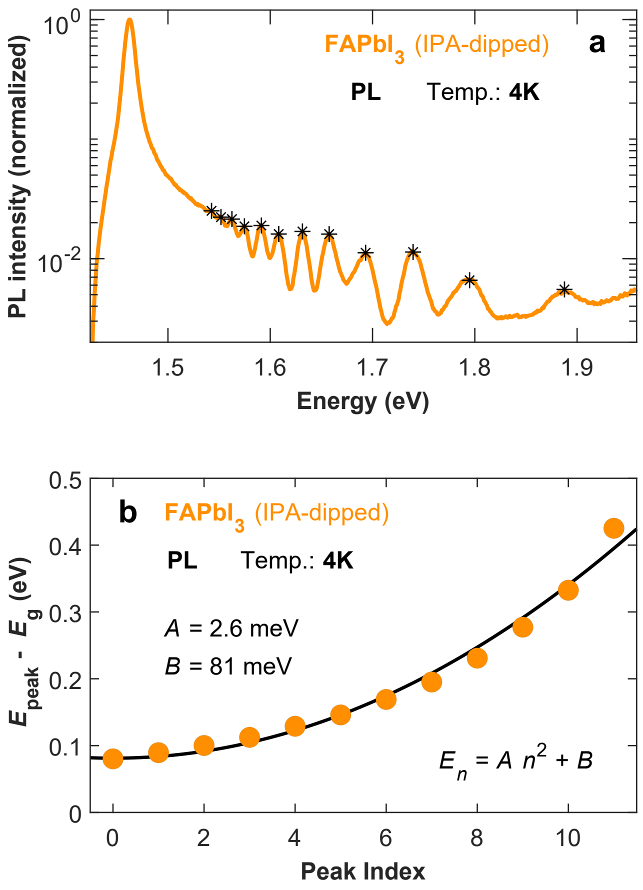

Supporting Figure S18: Confinement models for the infinite potential well using the PL peak features of IPA-dipped $FAPbI_3$. (**a**) shows the full photoluminescence spectrum of IPA-dipped $FAPbI_3$ at 4 K on a log scale, with the peaks of the PL features visually marked. (**b**) shows the quadratic fit to the PL confinement energies versus peak index, assuming a constant-sized quantum well.

6.2 $FA_{0.57}Cs_{0.43}PbI_3$

It is worth noting that for the low-temperature (4K) steady-state PL of the $FA_{0.57}Cs_{0.43}PbI_3$ film, two main PL peaks were observed (as can be seen in SI Figure Fig S19), where the higher intensity peak corresponds to the bandgap extracted from the absorption coefficient spectrum for the same film at 4K. This can be explained by bandtail recombination expected for metal-halide perovskites at low temperatures, as explained in previous work.[S28] The method we implemented of inducing partial substitution of FA with Cs both results in a spatial variation of the bandgap of the material across its thickness (i.e. a composition differential across the film exists), and creates comparatively a larger number of deep trap states than those present in the control film - corroborated by the reduction in PL amplitude with increasing Cs concentration present in the film (as displayed in Supporting Figure S5 and Supporting Figure S1 respectively). This creates a tail of deep trap states in the 43% Cs film, that would be difficult for the charge carriers to escape from with the limited thermal energy available at 4K. Additionally, at these low temperature, fewer phonons will be available to mediate non-radiative recombinations from sub-gap trap states, resulting in the emergence of a visible bandtail peak in the PL spectrum, as observed here. It has previously been claimed that bandtail emissions at low temperatures, photon recycling and reabsorption,[S29,S30] strong recombination due to excitons bound to surface defects,[S31] or segregation of the different compositions throughout the films (causing two main different compositions and PL peaks - especially for those films dipped in higher concentrations of cs-acetate) can cause the formation of two main PL peaks. We can first rule out excitonic emission, since the expected E_B from the PL peak separation does not match that obtained from the Elliott fits. Similarly photon recycling can be ruled out as a source for the second peak, because of its very limited influence on the PL spectrum of these 100 nm thin films - as discussed above. Although the prevalence of ionic motion in metal halide perovskites with temperature is complicated (owing to both the strength of entropic mixing and speed of ionic motion decreasing with a reduction in temperature),[S32] the absence of two onsets

or an additional feature in the absorption coefficient spectrum of this composition at 4K (displayed in Supporting Figure S10) can conclusively rule out cation segregation as an explanation. As a result, we conclude that the second PL peak arises from the existence of a tail of trap states causing prominent strong bandtail radiative recombination - as observed and explained before for metal-halide perovskites,[S28] and inorganic semiconductors.

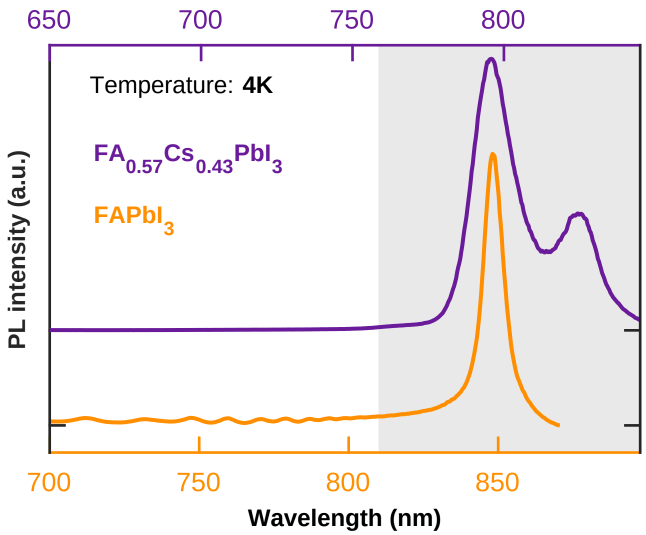

Supporting Figure S19: Photoluminescence spectra at 4 K of $FA_{1-x}Cs_xPbI_3$ films. Photoluminescence (PL) spectra of an $FAPbI_3$ (IPA dipped) and a $FA_{0.57}Cs_{0.43}PbI_3$ film at a temperature of 4 K showcasing the presence and absence, respectively, of above-bandgap peak features. The two spectra are vertically offset for visual clarity. The appearance of a second PL peak for the $FA_{0.57}Cs_{0.43}PbI_3$ film is due to the existence of a tail of deep trap states, which is further discussed in SI section 6.2

7 Time-resolved PL (TRPL)

TRPL of the thin films was measured using TCSPC (time-correlated single photon counting) following excitation by a 398 nm picosecond pulsed diode laser at a repetition rate of 1, 5, or 10 MHz (PicoHarp, LDH-D-C-405M), depending on the trace and to avoid wrap around and pile up. The resultant PL was collected and coupled into a grating spectrometer (Princeton Instruments, SP-2558), which directed the spectrally dispersed PL onto a photon-counting detector (PDM series from MPD), whose timing was controlled with a PicoHarp300 TCSPC event timer. The transients were recorded at the wavelengths corresponding to the main PL peak, and the observed PL peak features and valleys to probe the charge-carrier dynamics of the confined and unconfined regions.

The time-resolved PL traces of the $FAPbI_3$ (IPA-dipped) film at room temperature and the $FA_{0.57}Cs_{0.43}PbI_3$ film at 4 K are shown in Supporting Figures S20 and S21 respectively. The PL decay traces for both show a different dependence to what is observed for the decay traces of the PL peak features for the $FAPbI_3$ (IPA-dipped) film at 4 K.

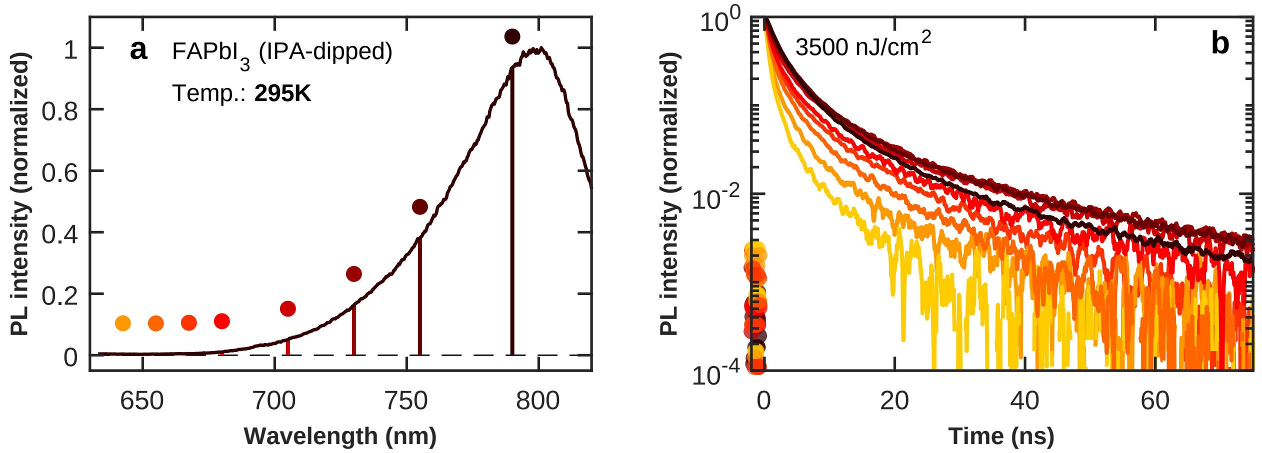

Supporting Figure S20: Steady-state and time-resolved photoluminescence of the IPA-dipped $FAPbI_3$ film at room temperature PL spectra of the IPA-dipped $FAPbI_3$ film at **a**: 295 K for the region expected to show high-energy peak features, and **b**: the corresponding time-resolved photoluminescence (TRPL) decay traces recorded at the wavelength of the peaks represented by the solid vertical lines and dots in (**a**).

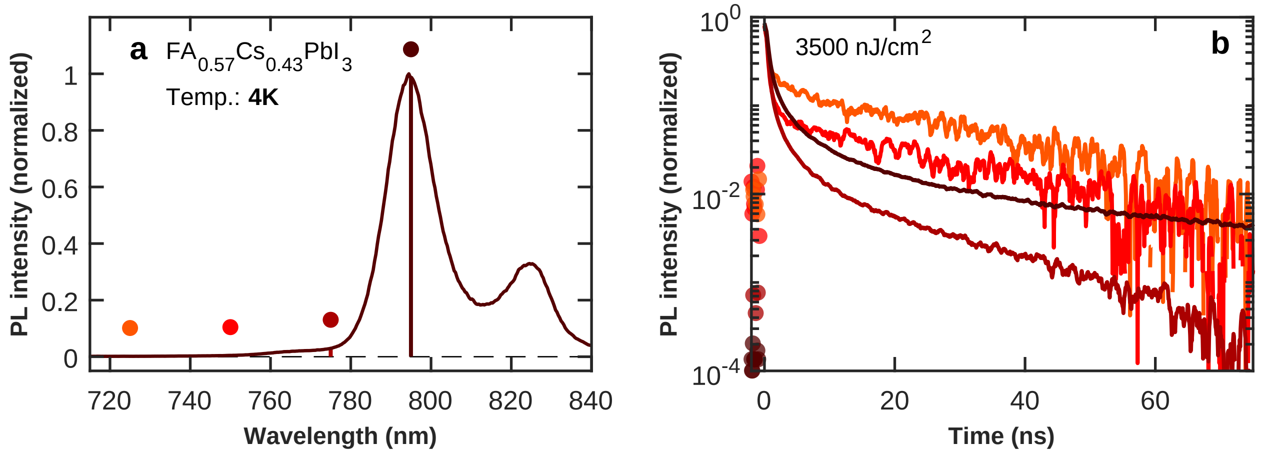

Supporting Figure S21: Steady-state and time-resolved photoluminescence for the $FA_{0.57}Cs_{0.43}PbI_3$ film at 4 K PL spectra of the $FA_{0.57}Cs_{0.43}PbI_3$ film at **a**: 4 K for the region expected to show high-energy peak features, and **b**: the corresponding time-resolved photoluminescence (TRPL) decay traces recorded at the wavelengths of the peaks represented by the solid vertical lines and dots in (**a**).

Instrument Response Function

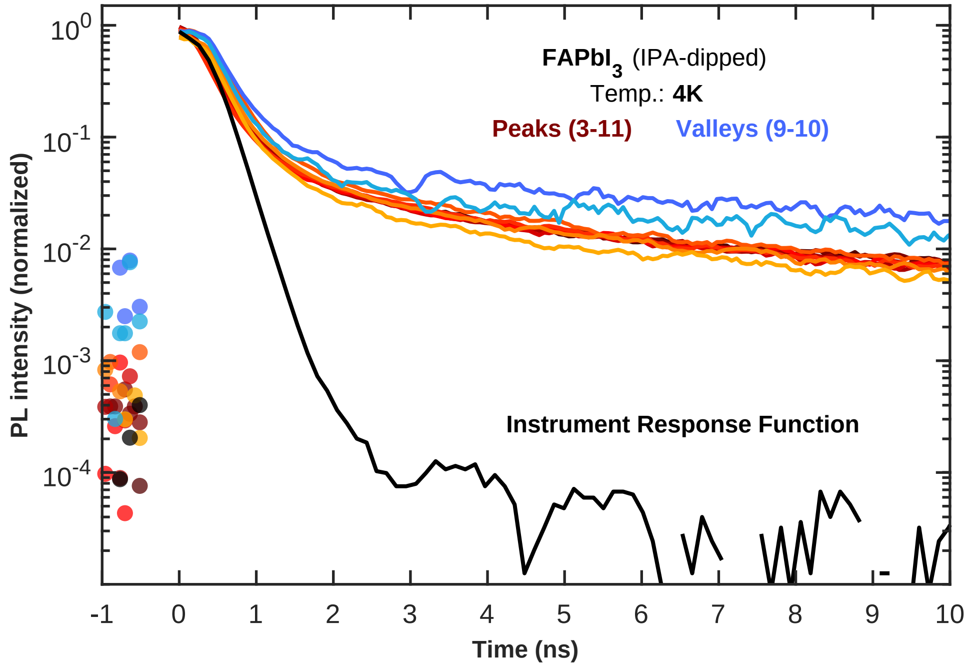

Supporting Figure S22: IRF and time-resolved PL decay traces for $FAPbI_3$ at early times after excitation. The IRF sets the intrinsic temporal resolution of the experimental setup being used in this study. The early-time similarity between the IRF trace and the PL decay traces make a distinction between the charge-carrier dynamics of the quantum-confined states (peaks) and the non-confined regions (valleys) difficult, especially at early time. It also prohibits time resolution of some of the relaxation pathways likely to occur at very early times, such as internal relaxation within the quantum well and its domains.

References

(S1) Jiang, Y.; Leyden, M. R.; Qiu, L.; Wang, S.; Ono, L. K.; Wu, Z.; Juarez-Perez, E. J.; Qi, Y. Combination of Hybrid CVD and Cation Exchange for Upscaling Cs-Substituted Mixed Cation Perovskite Solar Cells with High Efficiency and Stability. *Advanced Functional Materials* **2018**, *28*, 1703835.

(S2) Stoumpos, C. C.; Malliakas, C. D.; Kanatzidis, M. G. Semiconducting tin and lead iodide perovskites with organic cations: phase transitions, high mobilities, and near-infrared photoluminescent properties. *Inorganic Chemistry* **2013**, *52*, 9019–9038.

(S3) Trots, D.; Myagkota, S. High-temperature structural evolution of caesium and rubidium triiodoplumbates. *Journal of Physics and Chemistry of Solids* **2008**, *69*, 2520–2526.

(S4) Li, Z.; Yang, M.; Park, J.-S.; Wei, S.-H.; Berry, J. J.; Zhu, K. Stabilizing perovskite structures by tuning tolerance factor: formation of formamidinium and cesium lead iodide solid-state alloys. *Chemistry of Materials* **2016**, *28*, 284–292.

(S5) Weber, O. J.; Ghosh, D.; Gaines, S.; Henry, P. F.; Walker, A. B.; Islam, M. S.; Weller, M. T. Phase behavior and polymorphism of formamidinium lead iodide. *Chemistry of Materials* **2018**, *30*, 3768–3778.

(S6) Constantinos, S.; Christos, M.; Mercouri, K., *et al.* Semiconducting tin and lead iodide perovskites with organic cations: phase transitions, high mobilites and near-infrared photoluminescent properties. *Inorg. Chem* **2013**, *52*, 9019–9038.

(S7) Ghosh, D.; Smith, A. R.; Walker, A. B.; Islam, M. S. Mixed A-cation perovskites for solar cells: atomic-scale insights into structural distortion, hydrogen bonding, and electronic properties. *Chemistry of Materials* **2018**, *30*, 5194–5204.

(S8) Lee, J.-W.; Kim, D.-H.; Kim, H.-S.; Seo, S.-W.; Cho, S. M.; Park, N.-G. Formamidinium and cesium hybridization for photo-and moisture-stable perovskite solar cell. *Advanced Energy Materials* **2015**, *5*, 1501310.

(S9) Filip, M. R.; Eperon, G. E.; Snaith, H. J.; Giustino, F. Steric engineering of metal-halide perovskites with tunable optical band gaps. *Nature Communications* **2014**, *5*, 1–9.

(S10) Sutton, R. J.; Filip, M. R.; Haghighirad, A. A.; Sakai, N.; Wenger, B.; Giustino, F.; Snaith, H. J. Cubic or orthorhombic? Revealing the crystal structure of metastable black-phase $CsPbI_3$ by theory and experiment. *ACS Energy Letters* **2018**, *3*, 1787–1794.

(S11) Elliott, R. Intensity of optical absorption by excitons. *Physical Review* **1957**, *108*, 1384.

(S12) Davies, C. L.; Borchert, J.; Xia, C. Q.; Milot, R. L.; Kraus, H.; Johnston, M. B.; Herz, L. M. Impact of the organic cation on the optoelectronic properties of formamidinium lead triiodide. *The journal of physical chemistry letters* **2018**, *9*, 4502–4511.

(S13) Wright, A. D.; Volonakis, G.; Borchert, J.; Davies, C. L.; Giustino, F.; Johnston, M. B.; Herz, L. M. Intrinsic quantum confinement in formamidinium lead triiodide perovskite. *Nature Materials* **2020**, *19*, 1201–1206.

(S14) Galkowski, K.; Mitioglu, A.; Miyata, A.; Plochocka, P.; Portugall, O.; Eperon, G. E.; Wang, J. T.-W.; Stergiopoulos, T.; Stranks, S. D.; Snaith, H. J., *et al.* Determination of the exciton binding energy and effective masses for methylammonium and formamidinium lead tri-halide perovskite semiconductors. *Energy & Environmental Science* **2016**, *9*, 962–970.

(S15) Yang, Z.; Surrente, A.; Galkowski, K.; Miyata, A.; Portugall, O.; Sutton, R.; Haghighirad, A.; Snaith, H.; Maude, D.; Plochocka, P., *et al.* Impact of the halide cage on

the electronic properties of fully inorganic cesium lead halide perovskites. *ACS Energy Letters* **2017**, *2*, 1621–1627.

(S16) Masi, S.; Gualdrón-Reyes, A. F.; Mora-Sero, I. Stabilization of Black Perovskite Phase in $FAPbI_3$ and $CsPbI_3$. *ACS Energy Letters* **2020**, *5*, 1974–1985.

(S17) Zanatta, A. Revisiting the optical bandgap of semiconductors and the proposal of a unified methodology to its determination. *Scientific reports* **2019**, *9*, 1–12.

(S18) Prasanna, R.; Gold-Parker, A.; Leijtens, T.; Conings, B.; Babayigit, A.; Boyen, H.-G.; Toney, M. F.; McGehee, M. D. Band gap tuning via lattice contraction and octahedral tilting in perovskite materials for photovoltaics. *Journal of the American Chemical Society* **2017**, *139*, 11117–11124.

(S19) Davies, C. L.; Filip, M. R.; Patel, J. B.; Crothers, T. W.; Verdi, C.; Wright, A. D.; Milot, R. L.; Giustino, F.; Johnston, M. B.; Herz, L. M. Bimolecular recombination in methylammonium lead triiodide perovskite is an inverse absorption process. *Nature Communications* **2018**, *9*, 1–9.

(S20) Herz, L.; Phillips, R.; Le Ru, E.; Murray, R. Time-Resolved Photoluminescence Cross-Correlation Measurements on InAs Quantum Dots. *physica status solidi (a)* **2002**, *190*, 565–569.

(S21) Heinrichsdorff, F.; Grundmann, M.; Stier, O.; Krost, A.; Bimberg, D. Influence of In/Ga intermixing on the optical properties of InGaAs/GaAs quantum dots. *Journal of crystal growth* **1998**, *195*, 540–545.

(S22) Brunner, K.; Bockelmann, U.; Abstreiter, G.; Walther, M.; Böhm, G.; Tränkle, G.; Weimann, G. Photoluminescence from a single GaAs/AlGaAs quantum dot. *Physical review letters* **1992**, *69*, 3216.

(S23) Goni, A.; Born, H.; Heitz, R.; Hoffmann, A.; Thomsen, C.; Heinrichsdorff, F.; Bimberg, D. Magnetoluminescence study of annealing effects on the electronic structure of self-organized InGaAs/GaAs quantum dots. *Japanese Journal of Applied Physics* **2000**, *39*, 3907.

(S24) Sosnowski, T.; Norris, T.; Jiang, H.; Singh, J.; Kamath, K.; Bhattacharya, P. Rapid carrier relaxation in $In_{0.4}Ga_{0.6}As$/GaAs quantum dots characterized by differential transmission spectroscopy. *Physical Review B* **1998**, *57*, R9423.

(S25) Reed, M.; Bate, R.; Bradshaw, K.; Duncan, W.; Frensley, W.; Lee, J.; Shih, H. Spatial quantization in GaAs–AlGaAs multiple quantum dots. *Journal of Vacuum Science & Technology B: Microelectronics Processing and Phenomena* **1986**, *4*, 358–360.

(S26) Urayama, J.; Norris, T. B.; Singh, J.; Bhattacharya, P. Observation of phonon bottleneck in quantum dot electronic relaxation. *Physical review letters* **2001**, *86*, 4930.

(S27) Jia, X.; Jiang, J.; Zhang, Y.; Qiu, J.; Wang, S.; Chen, Z.; Yuan, N.; Ding, J. Observation of enhanced hot phonon bottleneck effect in 2D perovskites. *Applied Physics Letters* **2018**, *112*, 143903.

(S28) Wright, A. D.; Milot, R. L.; Eperon, G. E.; Snaith, H. J.; Johnston, M. B.; Herz, L. M. Band-Tail Recombination in Hybrid Lead Iodide Perovskite. *Advanced Functional Materials* **2017**, *27*, 1700860.

(S29) Schötz, K.; Askar, A. M.; Peng, W.; Seeberger, D.; Gujar, T. P.; Thelakkat, M.; Köhler, A.; Huettner, S.; Bakr, O. M.; Shankar, K., *et al.* Double peak emission in lead halide perovskites by self-absorption. *Journal of Materials Chemistry C* **2020**, *8*, 2289–2300.

(S30) Chen, Y.; Wang, T.; Li, Z.; Li, H.; Ye, T.; Wetzel, C.; Li, H.; Shi, S.-F. Communicating two states in perovskite revealed by time-resolved photoluminescence spectroscopy. *Scientific Reports* **2018**, *8*, 1–7.

(S31) Priante, D.; Dursun, I.; Alias, M. S.; Shi, D.; Melnikov, V.; Ng, T. K.; Mohammed, O. F.; Bakr, O.; Ooi, B. S. The recombination mechanisms leading to amplified spontaneous emission at the true-green wavelength in $CH_3NH_3PbBr_3$ perovskites. *Applied Physics Letters* **2015**, *106*, 081902.

(S32) Chen, T.; Foley, B. J.; Park, C.; Brown, C. M.; Harriger, L. W.; Lee, J.; Ruff, J.; Yoon, M.; Choi, J. J.; Lee, S.-H. Entropy-driven structural transition and kinetic trapping in formamidinium lead iodide perovskite. *Science advances* **2016**, *2*, e1601650.
